# Benzothiadiazole-based rotation and possible antipolar order in carboxylate-based metal-organic frameworks

**DOI:** 10.1038/s42004-023-00959-6

**Published:** 2023-07-29

**Authors:** Jennifer Schnabel, Arthur Schulz, Peter Lunkenheimer, Dirk Volkmer

**Affiliations:** 1 Chair of Solid State and Materials Chemistry, University of Augsburg, Institute of Physics, Universitaetsstrasse 1, 86159 Augsburg, Germany; 2 Experimental Physics V, Center for Electronic Correlations and Magnetism, University of Augsburg, Institute of Physics, Universitaetsstrasse 1, 86159 Augsburg, Germany

**Keywords:** Metal-organic frameworks, Computational methods, Computational chemistry, Chemical physics

## Abstract

By modifying organic ligands of metal-organic framework with dipolar units, they turn suitable for various applications, e.g., in the field of sensor systems or switching of gas permeation. Dipolar linkers in the organic ligand are capable to rotate in certain temperature and frequency ranges. The copper-bearing paddlewheel shaped metal-organic frameworks ZJNU-40 and JLU-Liu30 possess such a polarizable dipole moment due to their benzothiadiazole moiety in the organic ligands. Here, we investigate the molecular rotor behavior of benzothiadiazole units of the two carboxylate-based MOFs by dielectric spectroscopy and computational simulation. Our dielectric results provide clear evidence for significant reorientational relaxation dynamics of these rotors, revealing various characteristics of glasslike freezing upon cooling. The calculated rotational energy barriers are consistent with experimentally determined barriers for single-dipole dynamics. Moreover, for JLU-Liu30 we find hints at antipolar ordering below about 300 K.

## Abstract (web summary)

Metal–organic frameworks containing rotatable dipolar linkers are of interest for sensor systems and in gas separation. Here, the authors investigate the molecular rotor behaviour of benzothiadiazole units in the carboxylate-based MOFs ZJNU-40 and JLU-Liu30 using dielectric spectroscopy and computational simulations.

## Introduction

Metal-organic frameworks (MOFs) are a class of highly porous materials, which can be adapted to specific applications due to their high degree of tunability, structure diversity and chemical and physical properties^1^. The range of metal-organic frameworks has steadily expanded, and MOFs involving molecular machines are becoming increasingly interesting for electrical applications^2^, sensor technologies^3,4^, gas adsorption and storage^5^, and drug encapsulation^6^ or interactions with guest molecules^7^. By incorporating molecular dipolar units or even exploiting the design of rotational axes, internal dynamics in MOFs can be generated and introduced as a motor for rotation^8,9^. Influencing the electronic conjugations and plenty of free volume can also have an effect on the rotational dynamics^8,10–12^. Gonzalez-Nelson et al. have demonstrated that the linker alone can induce rotation in the MOF and that the rotation barrier can be reduced by functional groups^5^. This can also be accomplished by introducing strong dipolar moments as shown by Su et al. who even detected an antiferroelectric phase transition arising from the spontaneous ordering of the dipolar linkers^13^. Perego et al. revealed the utility of dipolar units as molecular machines that can respond to chemical or physical stimuli^8^. Here, we focus on the use of carboxylate-based MOFs with piezo active units, ZJNU-40^14^ and JLU-Liu30^15^. Both are copper-based NbO-type MOFs, isoreticular to NOTT-101^16^.

**Fig. 1:**
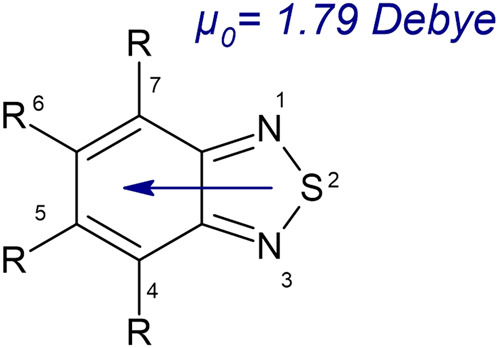
Lewis-structure of the 2,1,3-benzothiadiazole (BTD) moiety. The thiadiazole-moiety forms a permanent dipole moment of µ_0_ = 1.79 Debye with the remaining unit.

**Fig. 2:**
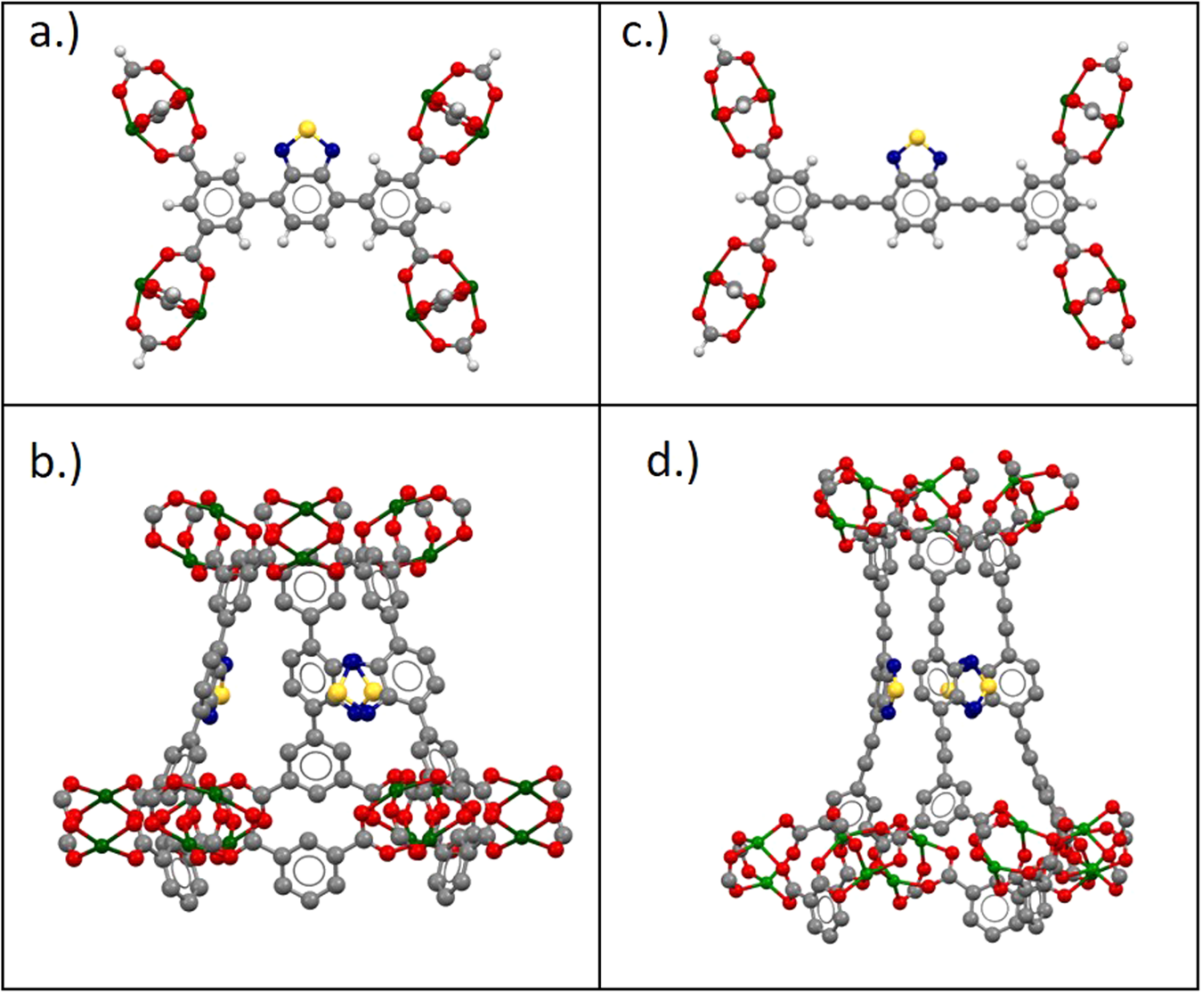
Description of the structures of ZJNU-40 and JLU-Liu30. **a** Cu-paddlewheel SBU of ZJNU-40 (**b**) view of SBU-unit of ZJNU-40 out of a-direction (**c**) Cu-paddlewheel SBU of JLU-Liu30 (**d**). view of SBU-unit of JLU-Liu30 out of a-direction; hydrogen is shown in white, oxygen in red, carbon in gray, copper in green, nitrogen in blue and sulfur in yellow.

Due to their electron-donating benzothiadiazole moiety in the carboxylate-based linker, these MOFs possess a dipole moment of 1.79 Debye^17,18^ (Fig. 1). The moiety can act as a rotor at certain frequencies and temperatures^19^. Moreover, applying a high electric field in principle can fix the direction of the macroscopic polarization. This enables a deliberate modification or even deformation of the lattice structure possible, which might be used for direction-oriented mass transport or sorting systems^14^. The robust Cu_2_(CO_2_)_4_-paddlewheel secondary building unit (SBU) of both MOFs allows one to engineer the activation energies of the rotational motion of the polar linker group to some extent^20^. The robust Cu_2_(CO_2_)_4_- paddlewheel (Fig. 2a, c) SBU is formed by two Cu^2+^ centres, linked to 4-bridged carboxylate linker. By connecting to further 3-connected carboxylate ligands (Fig. 2b, d), a three-dimensional framework is established. Both MOFs crystallize in the trigonal $R\bar{3}m$ space group. The two systems can be characterized with the *fof*-topology in Schönflies notation.

To comprehend the mechanism of molecular rotor, it is important to define its conceptualization. Kottas et al. defined a molecular rotor “as a system in which a molecule or part of a molecule rotates against another part of the molecule”^21^. The benzothiadiazole moiety is such a molecular rotor in the MOFs ZJNU-40 and JLU-Liu30 and single bond respectively the triple bond to the phenylene-units with the carboxylate-units can specify as the so-called axle^21,22^. It seems to be the main difference of the two MOFs: The linker of ZJNU-40, *5,5´-benzo[c][1,2,5]thiadiazole-4,7-diyldiisophthalic* acid (H_4_L), has simple C-C bonds in para-position of the benzo[1,2,5]thiadiazole-moiety as axle, whereas in JLU-Liu30 ethynyl groups (-C≡C-) of *5,5´-benzo[c][1,2,5]thiadiazole-4,7-diylbis(ethyne-2,1-diyl)diisophthalic* acid (H_4_btadpa) disassociate as rotary axis the benzothiadiazole ring and the phenyldicarboxylic acid groups. Thus, the bond type of the organic ligands alone can affect the intrinsic rotator barriers^10,21,23–25^. The triple bond is expected to result in extensive free rotation of the polar linker group and thus a lower rotational energy barrier in the rotor system than the single bonds^10,20,24^. In order to generate rotation of the molecular rotator by excitation with an E-Field, the rotational barrier of the rotator should be kept as low as possible. It has to be pointed out here that the dipolar motions in the investigated MOFs are not continuous but essentially correspond to flips between well-defined angles and several of such flips are necessary for a full 360° rotation.

Since both systems can be considered for electrical applications, we investigate the rotation barriers of the molecular rotors of both MOFs by different refined density functional theory (DFT) calculations and compare these with the experimentally obtained dielectric spectroscopy data in the following.

## Results and discussion

### Dielectric spectroscopy

**Fig. 3:**
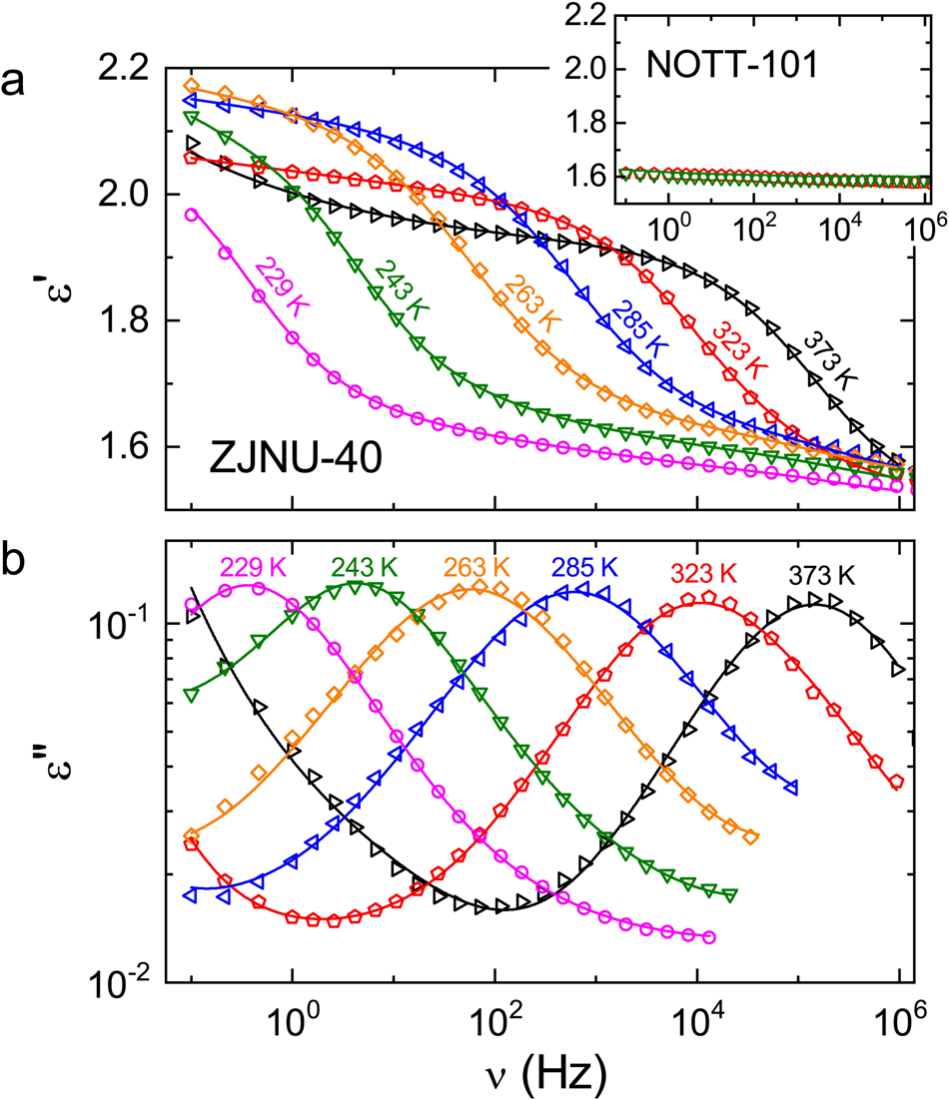
Dielectric permittivity spectra of ZJNU-40. The frequency-dependent dielectric constant *ε'* (**a**) and dielectric loss *ε"* (**b**) are shown for selected measuring temperatures. (At low temperatures, the loss spectra could not be measured up to the highest frequencies due to limitations in device resolution for the given sample geometry.) The lines are fits using the HN formula^28^ to model the dipolar relaxation features and additional contributions as explained in the text. They were simultaneously performed for the real and imaginary part. The inset shows *ε'*(*ν*) of the reference system NOTT-101, lacking any dipolar moment in its linkers, at two temperatures (243 and 323 K).

To obtain information on the reorientational dynamics and possible ordering phenomena of the molecular rotators of the individual carboxylate-based MOFs and to provide an estimate of the rotational barriers, the ligand dynamics were investigated using dielectric relaxation spectroscopy (DES)^13,17,20,25,26^. Figure 3 shows the frequency dependence of the dielectric constant *ε'* (a) and loss *ε"* (b) as measured for ZJNU-40 at various temperatures. It should be noted that the absolute values of both quantities only represent a lower limit, due to the reduced capacitor filling factor of powder samples compared to bulk samples. In general, the following conclusions are not affected by this fact. The *ε'*(*ν*) spectra (Fig. 3a) reveal a steplike feature and, at their point of inflection, *ε"*(*ν*) exhibits a peak (Fig. 3b). These are the typical indications of a so-called relaxation process, signifying reorientational fluctuations of dipolar entities^26^. In the present MOF, it can be ascribed to the rotational dynamics of the dipolar benzothiadiazole moieties in the linkers (Fig. 1). This is nicely corroborated by the inset of Fig. 3, showing *ε'*(*ν*) at 243 and 323 K for the reference system NOTT-101, which is isoreticular to ZJNU-40^14^ but lacks any dipolar moments of freely movable linker parts. In contrast to the *ε'* spectra of ZJNU-40 at these temperatures (main frame of Fig. 3a), the corresponding NOTT-101 spectra are featureless. Therefore, we conclude that the detected relaxation process found for ZJNU-40 indeed arises from dipolar rotational motions in its linkers. Both spectral relaxation features of ZJNU-40 in Fig. 3 shift to lower frequencies with decreasing temperature. As the loss peak frequency *ν*_p_ is inversely proportional to the relaxation time *τ*, characterizing the dipolar dynamics, this temperature-induced shift directly mirrors the slowing down of the dipolar motions upon cooling. The amplitude of the *ε'* step, corresponding to the so-called relaxation strength Δ*ε*, increases with decreasing temperature, which is typical for conventional dipolar relaxation processes^27^.

To gather quantitative information about the temperature-dependent relaxation time in ZJNU-40, the permittivity spectra of Fig. 3 were fitted using the often-applied empirical Havriliak-Negami (HN) function for the description of the dipolar relaxation features (lines)^28,29^. As revealed by Fig. 3, in addition to the bare peaks in *ε"*(*ν*) and steps in *ε'*(*ν*), there are numerous other spectral contributions: At first, at the high-frequency flank of the loss peaks and at low temperatures, *ε"*(*ν*) crosses over to a weaker frequency dependence, reminding of the onset of a secondary relaxation process located at higher-frequencies^30^. While the experimental resolution did not allow for an unequivocal detection of the corresponding secondary loss peak, for the spectra at *T* ≤ 322 K we formally accounted for this contribution by adding a second HN equation to the overall fit function. The occurrence of secondary relaxations, usually termed *β* relaxations, seems to be a universal feature of dipolar molecular liquids and glasses, but the underlying microscopic processes are still controversial^31–33^. Occasionally, they are also detected in plastic crystals^34^, materials exhibiting orientational degrees of freedom within the crystalline state, thus in some respect resembling the present MOFs. In the present case, however, we cannot fully exclude that reorientations of residual amounts of solvent molecules, which were occluded in the pores of the MOF framework during sample synthesis, could lead to the suggested secondary relaxation process, as was earlier found for MFU-4-type MOFs^35^. Nonetheless, a detailed treatment of this fast process is out of the scope of the present work, which concentrates on the dynamics of the dipolar linkers.

At the higher temperatures and at frequencies below the relaxation features, both *ε'*(*ν*) and *ε"*(*ν*) reveal an additional increase with decreasing frequency. This is typical for charge transport, triggered by the applied external electric field. We found that the assumption of a frequency-independent dc conductivity, *σ'* = *σ*_dc_, is insufficient to fit the spectra in this region: Via the general relation σ* = iε*ωε_0_ (with *ω* = 2*πν* and *ε*_0_ the permittivity of vacuum) between the complex conductivity σ* = *σ'* + *iσ"* and permittivity ε* = *ε'*−*iε"*, dc conductivity should lead to an 1/*ν* increase at low frequencies in *ε"*(*ν*) only. Instead, we partly had to assume additional ac conductivity to fit the spectra, which is commonly modeled by the so-called universal dielectric response (UDR) law^36^, a power law *σ'* = *σ*_*0* _*ν*^*s*^ with exponent *s* < 1. Via the Kramers-Kronig relation, this leads to a power law, *σ"* = tan(*sπ/2*)*σ*_*0*_*ν*^*s*^, in the imaginary part of the conductivity, too. Due to the relations *ε"* ∝ *σ'*/*ν* and *ε'* ∝ *σ"*/*ν*  (following from σ* = iε*ωε_0_) ac conductivity thus contributes to both the real and the imaginary part of the permittivity. UDR behavior is indicative of hopping charge transport of localized charge carriers as found in many kinds of disordered matter and in ionic conductors^37,38^. In the present case, we can only speculate about the nature of the charge carriers, but ions diffusing in tiny amounts of solvent at the surface of the powder grains is one possibility. Especially, Copper-paddlewheel units were demonstrated to undergo partial decomposition by a variety of pathways. Todaro et al. for instance have investigated the slow hydrolysis reaction of Copper-paddlewheel units in HKUST-1 at ambient conditions and they defined several models of defective paddlewheel units by means of ESR spectroscopy^39^. However, the time span during which slow decomposition was observed was about 20–50 days. Jeong et al. on the other hand demonstrated enhanced proton conductivity in HKUST-1, which was observed when methanol was coordinated to the open metal sites of the Cu(II) ions in the paddlewheel unit^40^. A study conducted by Friedländer et al. showed that MOFs containing paddlewheel units may contain a significant fraction of monomeric Cu(II) paddlewheel units^41^. Hence, there are several well-documented cases that demonstrate a certain structural variability of Copper-paddlewheel units under different conditions. Moreover, electrically conductive surface species might yield an additional contribution to this effect in general. Given the fact that the observed conductivity is lower than 10^−14^ Ω^−1 ^cm^−1^ even at the highest investigated temperature the estimated number of potential defect sites should be very small, which rules out the possibility to resolve the structural origin of this effect and it only shows up in the spectra due to the very high resolution of the employed dielectric devices.

Finally, we want to point out that the additional contributions, needed to fit the complete dielectric spectra of Fig. 3, do not have any significant effect on the parameters of the main relaxation feature. This is especially valid for the relaxation time, the main outcome of our analysis, which is well defined by the point of inflection in *ε'*(*ν*) and the peak frequency in *ε"*(*ν*), both clearly discernible in the respective spectra.

Compared to the half width of 1.14 decades predicted by the Debye theory^27^, which assumes exponential relaxation of independent dipoles, the peaks in Fig. 3b are significantly broadened. This is also confirmed by the fits with the HN function^28^ (lines in Fig. 3), whose parameters indicate a symmetric broadening for most temperatures. In general, a broadening of loss peaks, termed non-exponentiality, is a hallmark feature of supercooled glass-forming liquids and plastic crystals^34,42,43^ and commonly ascribed to a distribution of relaxation times due to heterogeneity^44,45^. In amorphous materials as glasses or liquids, this is simply caused by the structural disorder. However, in plastic crystals^34^ or other crystalline materials with dipolar reorientations, like certain MOFs^5,46^, such broadening is also commonly found, although they have a well-ordered crystalline lattice. There one can assume that the different environment, sensed by each dipole, is caused by interactions with the neighboring dipoles whose orientations fluctuate and are disordered. These interactions may, e.g., be of direct dipole-dipole nature or due to steric hindrance^34^. The resulting different environment for each dipole influences its energy barrier for reorientation and, thus, gives rise to a somewhat different relaxation time, explaining the peak broadening. Interlinker steric interactions were, e.g., recently found to explain the distribution of relaxation times detected in a MOF from the MIL-53 family^5^.

**Fig. 4:**
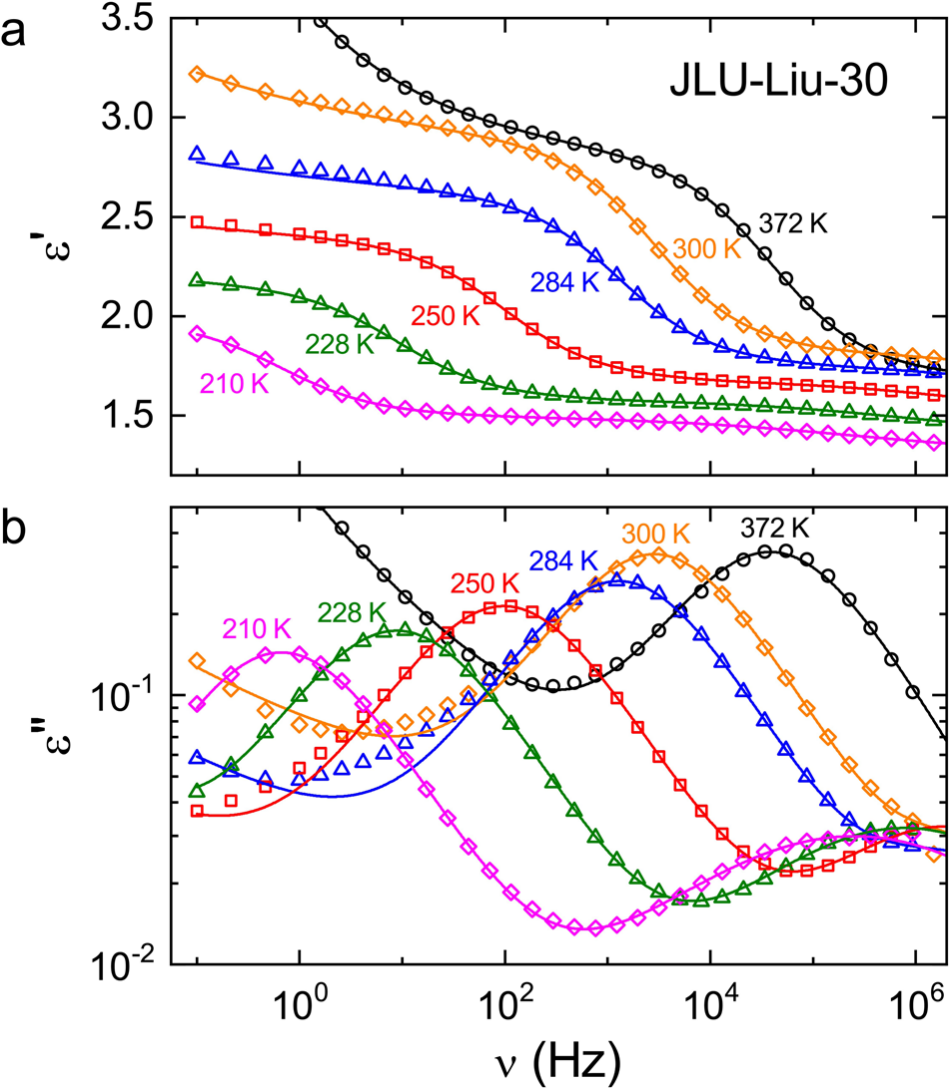
Dielectric permittivity spectra of JLU-Liu30. The frequency-dependent dielectric constant *ε'* (**a**) and dielectric loss *ε"* (**b**) are shown for selected measuring temperatures. The lines are simultaneous fits of the real and imaginary part using the HN formula for the main dipolar relaxation feature and additional contributions as explained in the text.

Figure 4 shows the permittivity spectra of JLU-Liu30, again revealing typical dipolar relaxation features. Additional contributions from charge transport and a secondary relaxation (more clearly resolved than in ZJNU-40) show up in the spectra. The lines are fits performed in the same way as for ZJNU-40. Some deviations between fits and experimental data, seen in the minimum region of *ε"*(*ν*) especially at 250–300 K, may indicate a minor additional relaxation process, but this does not affect the analysis of the main process. Just as for ZJNU-40, the loss peaks are symmetrically broadened, compared to the expectations for exponential single-particle relaxation, indicating heterogeneity that causes a distribution of relaxation times. In contrast to ZJNU-40, the amplitudes of the *ε'* and *ε"* relaxation features, which are proportional to the relaxation strength, decrease with decreasing temperature below about 300 K. Interestingly, such non-canonical behavior is often found in materials with polar order at temperatures below the polar phase-transition^47,48^. This finding will be treated in more detail below.

**Fig. 5:**
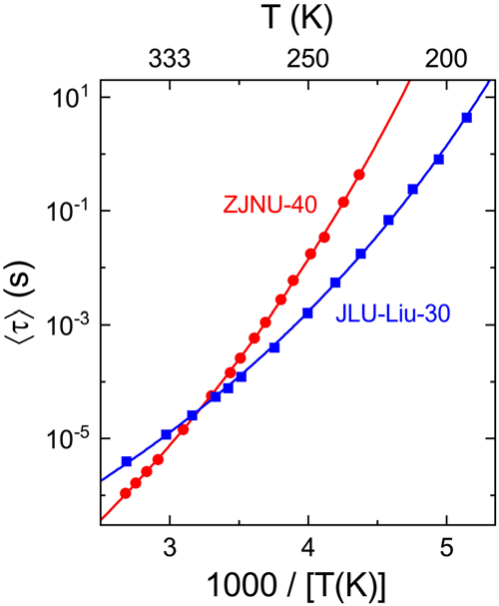
Temperature dependence of the relaxation times. The symbols indicate the mean relaxation times of both investigated MOFs as determined from the fits of their permittivity spectra (cf. Figs. 3 and 4). The lines are fits with the VFT function, Eq. (1), leading to *τ*_0_ = 7.8 × 10^−12^ s, *D* = 33.5 and *T*_VF_ = 97.1 K for ZJNU-40 and *τ*_0_ = 1.7 × 10^−9 ^s, *D* = 21.7 and *T*_VF_ = 97.2 K for JLU-Liu30.

Figure 5 presents the temperature dependence of the mean relaxation times as determined from the fits of the measured permittivity spectra (see Figs. 3 and 4 for examples at selected temperatures). For canonical thermal activation of the rotational motions, an Arrhenius law, 〈*τ*〉 ∝ exp[*E*/(*k*_B_*T*)], would be expected (*E* denotes the energy barrier). In the Arrhenius representation of Fig. 5, this should lead to linear behavior with a slope that is proportional to *E*. However, the experimental data clearly deviate from this prediction and exhibit a continuous curvature. This again is a characteristic feature of materials showing glassy freezing^42,43^. There, such non-Arrhenius temperature dependence is usually fitted by the empirical Vogel-Fulcher-Tammann (VFT) function, used here in its modified form as proposed by Angell:^49^

1 $$\left\langle \tau \right\rangle ={\tau }_{0}\exp \left[\frac{D{T}_{{{\mbox{VF}}}}}{T-{T}_{{{\mbox{VF}}}}}\right]$$

In this equation, *T*_VF_ is the Vogel-Fulcher temperature, where 〈*τ*〉 diverges, and *τ*_0_ can be regarded as inverse attempt frequency. *D* represents the so-called strength parameter, quantifying the deviations from Arrhenius behavior (large *D* means small deviations; see ref. ^49^ for details). The empirical VFT function was originally proposed for glass-forming supercooled liquids^50,51^. The corresponding increasing slope revealed in the Arrhenius plot with decreasing temperature (cf. Fig. 5), is nowadays quite commonly ascribed to an increase of the cooperativity of molecular motion when the glass transition is approached upon cooling^52,53^. Here the term "cooperativity" is used in the sense of the Adam-Gibbs theory of the glass transition^54^ and of newer theories expanding it^55–57^. It essentially means that the molecules "collectively rearrange over some length scale"^53^. The increasing cooperativity corresponds to an increase of this length scale, finally explaining the non-Arrhenius behavior^52–57^. The applicability of the VFT equation is also well established for systems showing glassy freezing of non-structural dynamics. Prominent examples are the plastic crystals mentioned above^34^, molecular materials where the molecules are located on a well-defined crystalline lattice but still exhibit reorientational dynamics. In many of these systems, upon cooling this dynamics reveals glassy freezing, i.e., instead of ordering at a phase transition, it continuously slows down over many decades^34^. Finally, it comes to an effective halt, forming a so-called glassy crystal below an orientational glass-transition temperature, defined by 〈*τ*〉(${T}_{g}^{o}$) ≈ 100 s. In plastic crystals, the deviations of the temperature-dependent relaxation time from Arrhenius behavior are usually not very pronounced^34,58^ but there are also some exceptions^59^. Overall, while we do not have a direct proof that the non-Arrhenius behavior detected in the investigated two MOFs is due to cooperativity, based on the current understanding of glassy freezing, it seems the best explanation.

As mentioned above, in some respects, the MOFs investigated in the present work resemble plastic crystals as they also comprise dipolar degrees of freedom within a crystalline material. Indeed, the VFT behavior of 〈*τ*〉(*T*) evidenced by Fig. 5 points to cooperativity between the rotating dipoles, just as in plastic crystals. From the deduced strength parameters (*D* = 33.5 for ZJNU-40 and *D* = 21.7 for JLU-Liu30), the so-called fragility *m* can be calculated^60^. It is the most common parameter for quantifying the non-Arrhenius behavior. The obtained values of *m* = 33.6 (ZJNU-40) and *m* = 43.2 (JLU-Liu30) signify only moderate deviations from Arrhenius temperature dependence, just as in most plastic crystals^34,59^. Both MOFs should also feature a glass transition with respect to their orientational dipolar dynamics. Using the definition 〈*τ*〉(${T}_{g}^{o}$) ≈ 100 s, the orientational glass temperature ${T}_{g}^{o}$ can be estimated from the VFT fits. We obtain 205 K for ZJNU-40 and 182 K for JLU-Liu30. Below these temperatures, the rotational motions essentially freeze in and a kind of "orientational glass" state with (nearly) static orientational disorder is reached.

The computational calculations presented in the next section provide estimates of the potential energy barriers of a single rotor unit in MOF ZJNU-40 and JLU-Liu30. As discussed above, in contrast the dielectric data reveal a temperature-dependent energy barrier which is strongly influenced by cooperative interactions between the dipoles^52,53^. These interactions, which can have different origins like direct dipole–dipole interactions or steric effects, are not accounted for by the calculations. To enable a comparison of the dielectric and computational results, the single-dipole energy barriers *E*_*s*_ that would be measured in absence of any cooperativity can be estimated from the parameters of the performed VFT fits of 〈*τ*〉(*T*): As mentioned above, cooperativity increases when approaching the glass transition upon cooling. Correspondingly, it decreases with increasing temperature and for *T* → ∞ it should vanish. For very high temperatures, non-cooperative single-dipole dynamics should be observed because there any type of interdipole interactions leading to cooperativity can be neglected, compared to the dominant thermal energy *k*_B_*T*. For *T* → ∞, Eq. (1) indeed crosses over into simple Arrhenius behavior with an energy barrier (in K) of *E*_*s*_ = *DT*_VF_. We thus obtain 27 kJ/mol and 17 kJ/mol for the single-dipole rotational energy barriers in ZJNU-40 and JLU-Liu30, respectively.

**Fig. 6:**
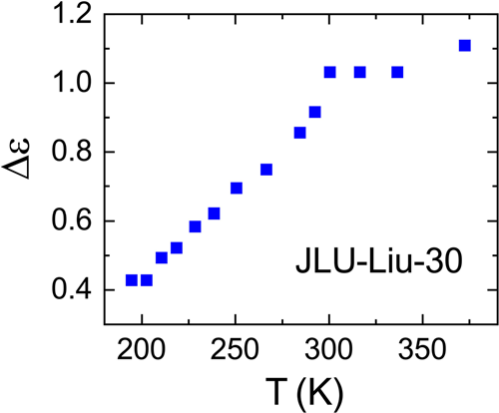
Dielectric strength of JLU-Liu30. The squares show the temperature dependence of Δ*ε* as obtained from the fits of the permittivity spectra.

**Fig. 7:**
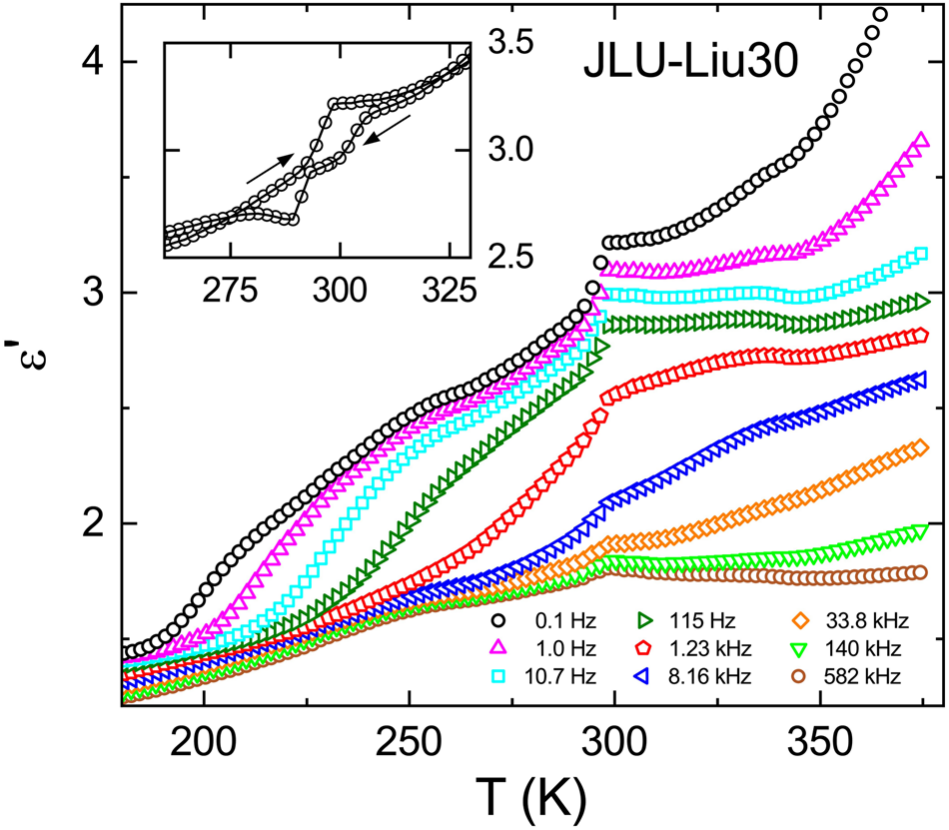
Temperature dependence of the dielectric constant of JLU-Liu30. The symbols show *ε'*(*T*) at various measurement frequencies as detected upon heating. The inset shows the heating and cooling curve for 0.1 Hz (the lines connect the data points).

Finally, we come back to the anomalous temperature dependence of the relaxation strength below about 300 K, indicated by the permittivity spectra of JLU-Liu30 (Fig. 4). Figure 6 shows Δ*ε*(*T*) as obtained from the fits of the permittivity spectra (Fig. 4). It reveals a clear crossover from weak temperature variation at *T* ≥ 300 K to a rather strong decrease for lower temperatures. This finding could indicate a phase transition to polar order below 300 K. The most direct check of polar phase transitions in dielectric spectroscopy is the inspection of the temperature-dependent dielectric-constant data which should exhibit an anomaly at the transition temperature. Figure 7 presents temperature-dependent *ε'* data as measured upon heating at various frequencies. In this plot, the detected dipolar relaxation process of JLU-Liu30 (Fig. 4) is revealed by steps from low to high values of *ε'* that shift to higher temperatures with increasing frequency (this trivially follows from the occurrence of relaxation steps in the *ε'*(*ν*) spectra, shifting to higher frequencies with increasing temperature, cf. Figure 4a). Interestingly, superimposed to these features, there is a significant anomaly in *ε'*(*T*) at about 295 K. For a phase transition, a corresponding anomaly should also be revealed upon cooling. As shown in the inset of Fig. 7, presenting the cooling and heating curves for 0.1 Hz as an example, this indeed is the case. However, upon cooling two successive anomalies are observed, separated by about 10 K, for which we currently have no explanation.

At a ferroelectric order transition, leading to parallel arrangement of the dipoles, *ε'*(*T*) usually shows a well-pronounced peak at the transition temperature *T*_c_ (refs. ^47,48^) in contrast to the primarily steplike anomaly observed in Fig. 7. In so-called order-disorder ferroelectrics, where the dipoles already exist above the transition, dielectric spectroscopy reveals significant dipolar relaxation dynamics both above and below *T*_c_, just as in the present case^47,48^. However, below the transition the relaxation times should decrease with decreasing temperature, again at variance with the present findings (cf. Fig. 5). Overall, the current results are incompatible with ferroelectric ordering. A second possibility is antiferroelectric polar order. (Here we use the term “antiferroelectric” to denote antiparallel dipole order. It is important to point out that the definition of antiferroelectricity sometimes also includes switchability of the polarization, which was not tested in the present powder sample.) Indeed, a steplike anomaly as observed in Fig. 7 is in accord with theoretical predictions for *ε'*(*T*) at antiferroelectric phase transitions^61,62^. Interestingly, the cyanides KCN and NaCN show very similar *ε'*(*T*) behavior around their antiferroelectric transitions as JLU-Liu30^63^. These are well-known crystalline materials with reorientational degrees of freedom, just as in the present MOF. In both cyanides, the dumbbell-shaped CN^−^ ions undergo reorientational motions at high temperatures and exhibit antiferroelectric order at low temperatures^63^. For instance similar steplike *ε'*(*T*) anomalies have been recently found for several antipolar lacunar spinels as well^64,65^. Moreover, all these antipolar materials exhibit significant relaxational dynamics in the ordered state^63–65^. Just as for order-disorder ferroelectrics^47,48^, the relaxational dynamics below *T*_c_ in antiferroelectrics can be assumed to arise from dipoles that do not participate in the polar order. It seems reasonable that such dipoles are most numerous just below the transition. Correspondingly, the decrease of the relaxation strength, reported for KCN and NaCN below *T*_c_ in ref. ^63^ was stated to reflect "the gradual disappearance of alignable dipoles due to the onset of a second-order phase transition into the antiferroelectric ordered state". The same effect can be assumed to explain the reduction of Δ*ε* in the present case (Fig. 6). If there is an antiferroelectric phase transition in JLU-Liu30 at about 300 K, it seems puzzling that the dipolar relaxation times 〈*τ*〉(*T*) shown in Fig. 5 do not exhibit any significant anomaly at this temperature. Unfortunately, in literature there is only sparse information on the dipolar dynamics above and below the phase transition of antiferroelectrics. However, in the cyanides KCN and NaCN as well as in the lacunar spinel GaNb_4_S_4_, where *τ*(*T*) data are available^66,67^, interestingly there are no indications for a significant anomaly in 〈*τ*〉(*T*), too. We also tried to detect this suggested phase transition by DSC measurements but did not find any significant anomalies. However, one should be aware that in this MOF the ordering dipolar entities represent only a small fraction of the overall structure. Finally, we want to remark that our dielectric data cannot provide an absolute proof for antiferroelectric ordering, but we think at least there are strong hints at such order in this MOF.

### Torsion potential calculations

**Fig. 8:**
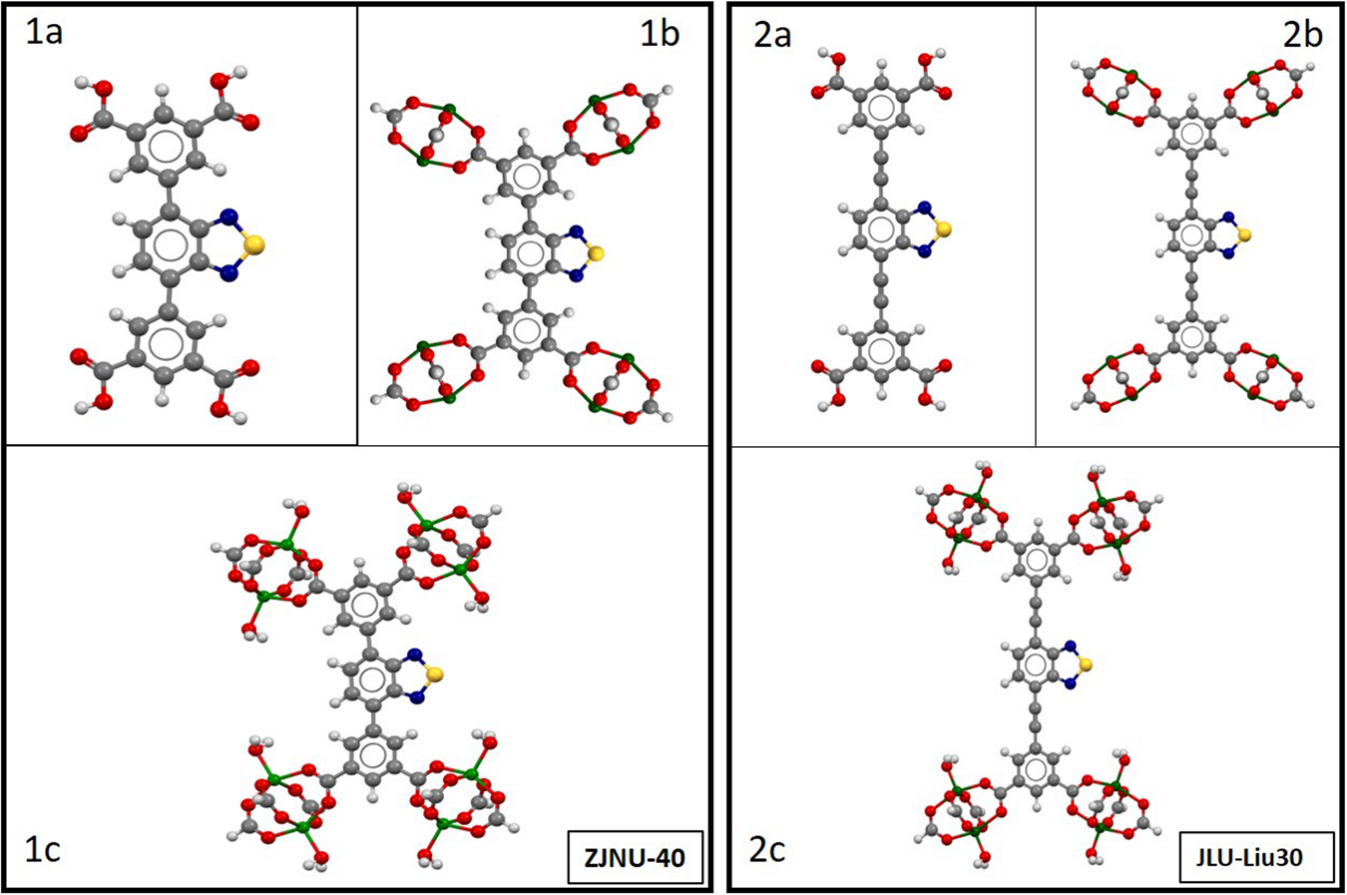
Initial geometries of molecular rotors representing excerpts from the 3D crystal lattices of ZJNU-40 (1) and JLU-Liu30 (2). (**1a**) schematic illustration of the simple linker of ZJNU-40, (**1b**) schematic illustration consisting of simple linker unit of ZJNU-40 with four Copper-paddlewheels moieties (**1c**) schematic overview of the molecular rotor unit with the presence of water coordinated to the metal ions in the paddlewheel units of ZJNU-40, (**2a**) schematic illustration of the simple linker of JLU-Liu30, (**2b**) schematic illustration consisting of simple linker unit of JLU-Liu30 with four Copper- paddlewheels moieties, (**2c**) schematic overview of the molecular rotor unit with the presence of water coordinated to the metal ions in the paddlewheel units of JLU-Liu30.

In order to correlate results from dielectric spectroscopy with simulations, calculations at different theoretical levels were performed. At the first approximation level, molecular complexes of different sizes, all comprising a single rotor, have been constructed, which are shown in Fig. 8.

**Fig. 9:**
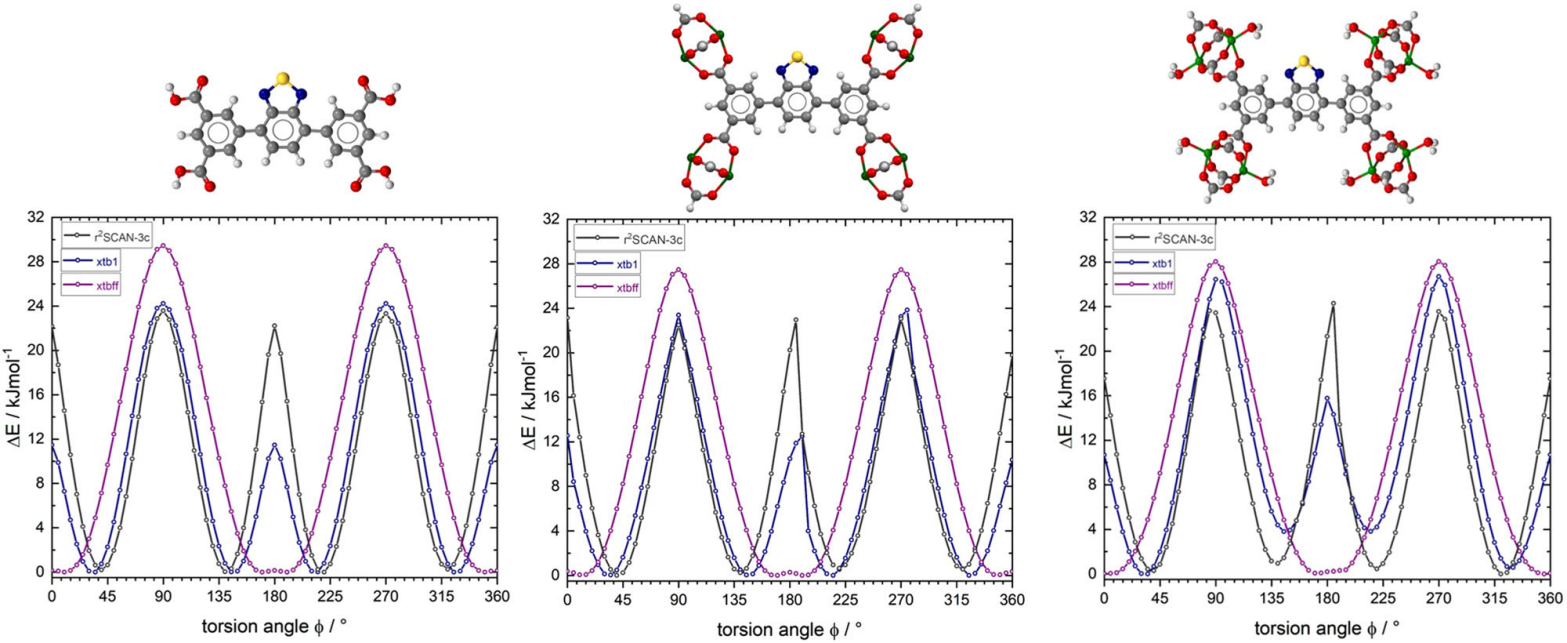
Torsion potential curves for rotor-stator model compounds of ZJNU-40 related to the fragments 1a-c. The dark gray curves represent the torsion potential calculation of r^2^SCAN-3c, violet represents the torsional motion of GFN-FF method, and in blue the calculated motion of semi-empirical GFN-xtb1.

**Fig. 10:**
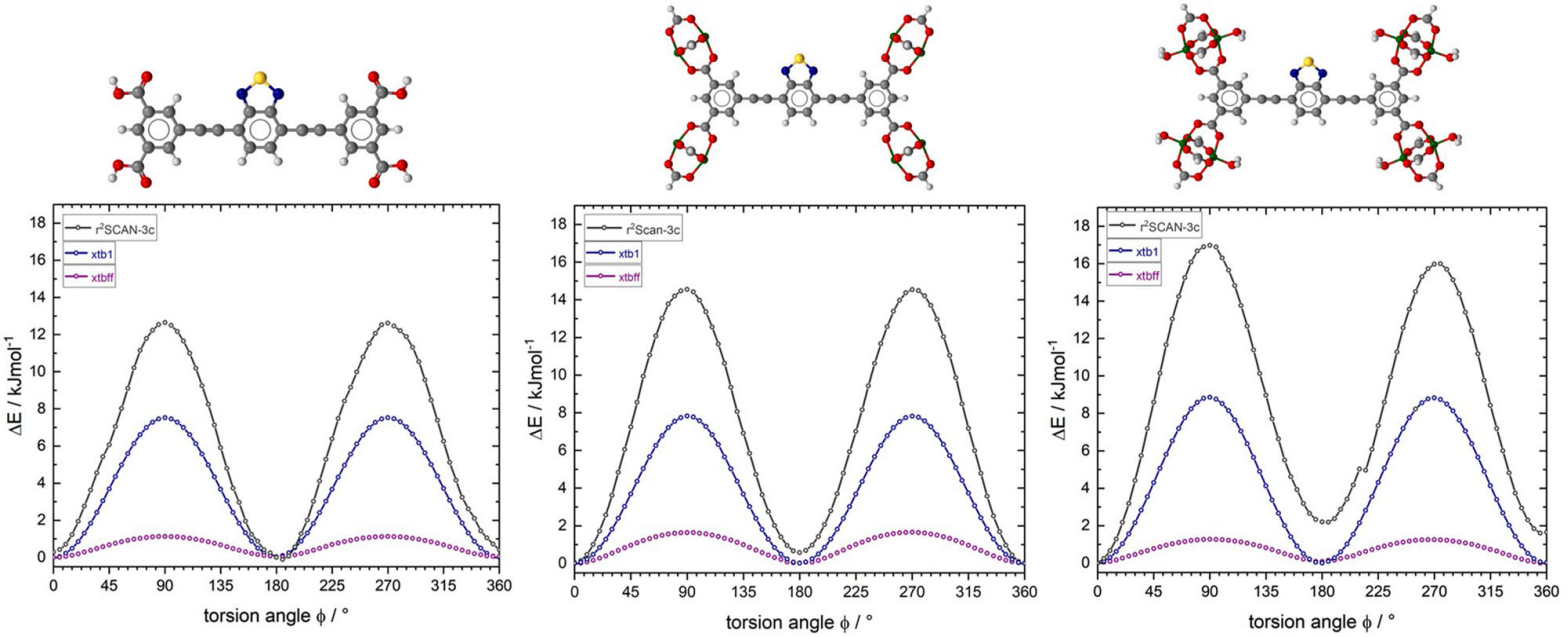
Torsion potential curves for rotor-stator model compounds of JLU-Liu30 related to the fragments 2a-c. The dark gray curves represent the torsion potential calculation of r^2^SCAN-3c, violet represents the torsional motion of GFN-FF method, and in blue the calculated motion of semi-empirical GFN-xtb1.

In order to estimate the validity and accuracy of different theoretical levels, each potential scan has been performed with a molecular mechanic, a semi-empirical, and a density functional theoretical approach. For molecular mechanics calculations, we chose the newly developed automated partially polarizable generic force-field („GFN-FF“)^68^, whereas for semiempirical calculations the tight-binding quantum chemical method „GFN-xtb1“ (with D3 dispersion correction)^69^, was employed. Ab initio DFT calculations were performed with a recently developed meta-generalized-gradient approximation (mGGA) functional r^2^SCAN-3c (with D4 dispersion correction and geometrical counter-poise corrections for London-dispersion and basis set superposition error)^69^. All three methods have been developed and parametrized by the working group of Grimme et al. thus enabling a consistent scheme of increasing accuracy for predicting activation energy values for the full rotation of the dipolar rotors with respect to their stators. The selection of these methods was partially gathered from a general discussion of best practice DFT protocols for basic molecular computational chemistry, as reported in ref. ^70^. However, opt-in for the GFN-xtb1(-D3) as opposed to the more robust GFN-xtb-2(-D4) approach was gleaned by the fact that all three theoretical methods should also be available for performing calculations under 3D periodic boundary conditions, which was not available for GFN-xtb-2 at the time of conducting these studies. Geometrical constraints on internal dihedral (=torsion) angles of the molecular fragments have been employed such as the dipolar rotor (=the benzothiadiazole moiety) rotates between two stators, the positions of the latter were held constrained within a common plane. The twisting motion of the rotor was scanned at steps of 5 degree for a full turn (360°). Each rotamer configuration was started from the same reference state. This procedure provides a first approximation of the potential energy of a single rotor unit in MOF ZJNU-40^14^ and JLU-Liu30^15^, respectively. The latter framework contains a rotor interspersed between triple bonds. Molecular fragments of increasing size have been constructed in order to estimate the influence of functional groups, presence of metal ions (paddlewheel units!) and the presence of water coordinated to the metal ions in the paddlewheel units. The results of the torsion scans are plotted in Figs. 9 and 10, correspondingly.

Activation energy parameters gleaned from these calculations are summarized in Table 1.

**Table 1:** Activation energy parameters for rotor-stator model compounds as discussed in the text based on the torsion potential calculation of ZJNU-40 and JLU-Liu30 .

ZJNU-40	GFN-FF	GFN-xtb1	r^2^SCAN-3c
**1a**	φ = 10° ΔE_min_ = 0.00 kJ/mol	φ = 35° ΔE_min_ = 0.00 kJ/mol	φ = 40° ΔE_min_ = 0.19 kJ/mol
	φ = 90° ΔE_max_ = 29.44 kJ/mol	φ = 90° ΔE_max_ = 24.22 kJ/mol	φ = 90° ΔE_max_ = 23.58 kJ/mol
**1b**	φ = 15° ΔE_min_ = 0.00 kJ/mol	φ = 35° ΔE_min_ = 0.00 kJ/mol	φ = 40° ΔE_min_ = 0.00 kJ/mol
	φ = 90° ΔE_max_ = 27.46 kJ/mol	φ = 90° ΔE_max_ = 23.38 kJ/mol	φ = 90° ΔE_max_ = 22.50 kJ/mol
**1c**	φ = 5° ΔE_min_ = 0.05 kJ/mol	φ = 35° ΔE_min_ = 0.00 kJ/mol	φ = 40° ΔE_min_ = 0.29 kJ/mol
	φ = 90° ΔE_max_ = 28.03 kJ/mol	φ = 90° ΔE_max_ = 26.45 kJ/mol	φ = 85° ΔE_max_ = 23.62 kJ/mol

JLU-Liu30	GFN-FF	GFN-xtb1	r^2^SCAN-3c
**2a**	φ = 0° ΔE_min_ = 0.00 kJ/mol	φ = 0° ΔE_min_ = 0.00 kJ/mol	φ = 0° ΔE_min_ = 0.28 kJ/mol
	φ = 90° ΔE_max_ = 1.13 kJ/mol	φ = 90° ΔE_max_ = 7.52 kJ/mol	φ = 90° ΔE_max_ = 12.64 kJ/mol
**2b**	φ = 0° ΔE_min_ = 0.0 kJ/mol	φ = 0° ΔE_min_ = 0.0 kJ/mol	φ = 0° ΔE_min_ = 0.0 kJ/mol
	φ = 90° ΔE_max_ = 1.64 kJ/mol	φ = 90° ΔE_max_ = 7.82 kJ/mol	φ = 90° ΔE_max_ = 14.55 kJ/mol
**2c**	φ = 0° ΔE_min_ = 0.0 kJ/mol	φ = 0° ΔE_min_ = 0.0 kJ/mol	φ = 0° ΔE_min_ = 0.0 kJ/mol
	φ = 90° ΔE_max_ = 1.27 kJ/mol	φ = 90° ΔE_max_ = 8.86 kJ/mol	φ = 90° ΔE_max_ = 16.98 kJ/mol

### Rotors mounted between single bonds (ZJNU-40)

All three methods predict similar maximum potential energy barriers for the flips of the rotor between different angles with respect to its stator (~24 – 30 kJ/mol). Neither the size of the molecular fragment, nor the presence of water molecules coordinated to the metal ions of the paddlewheel units has a major influence on the calculated energies. However, GFN-FF yields incorrect full torsion potentials for biphenyl type aromatic systems. The coplanar arrangement of aromatic rings is energetically favored for such systems, yielding incorrect (far too low) energies for such rotamers (Fig. 10). A similar but less pronounced trend is seen in GFN-xtb1(-D3) calculations. However, the qualitative and quantitative matching of the calculated potential energy values in comparison to the far more accurate r^2^SCAN-3c DFT calculations is promising taking into account of 3D periodic MD calculations on MOF unit cells (and super cells), which are intractable with DFT calculations.

From the experimental results of the DES-measurements, we determined a rotational barrier of 27 kJ/mol by the Vogel-Fulcher-Tammann approximation. By the GFN-FF method in particular, we found good agreement of the calculated rotational barriers of the individual molecular fragments with the determined data.

### Rotors mounted between triple bonds (JLU-Liu30)

**Fig. 11:**
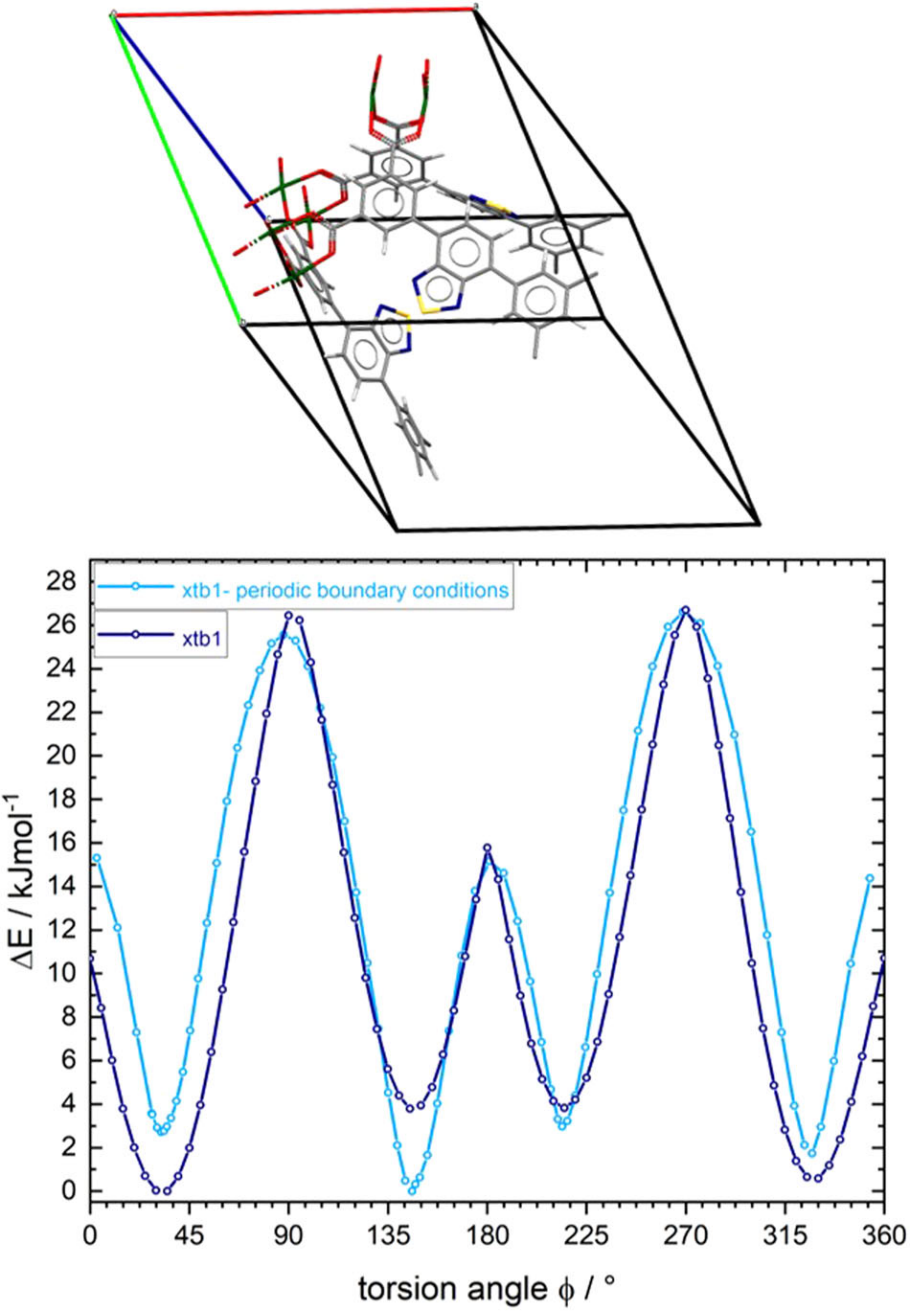
Comparison of calculated torsional potential curves considering 3D boundary conditions of ZJNU-40. GFN-xtb1 calculated torsion potential curves for the rotation of a single benzothiadiazole rotor placed in cluster compound **1c** (cf. Fig. 8; dark-blue curve) and in the primitive unit cell of ZJNU-40 (light blue curve), respectively.

**Fig. 12:**
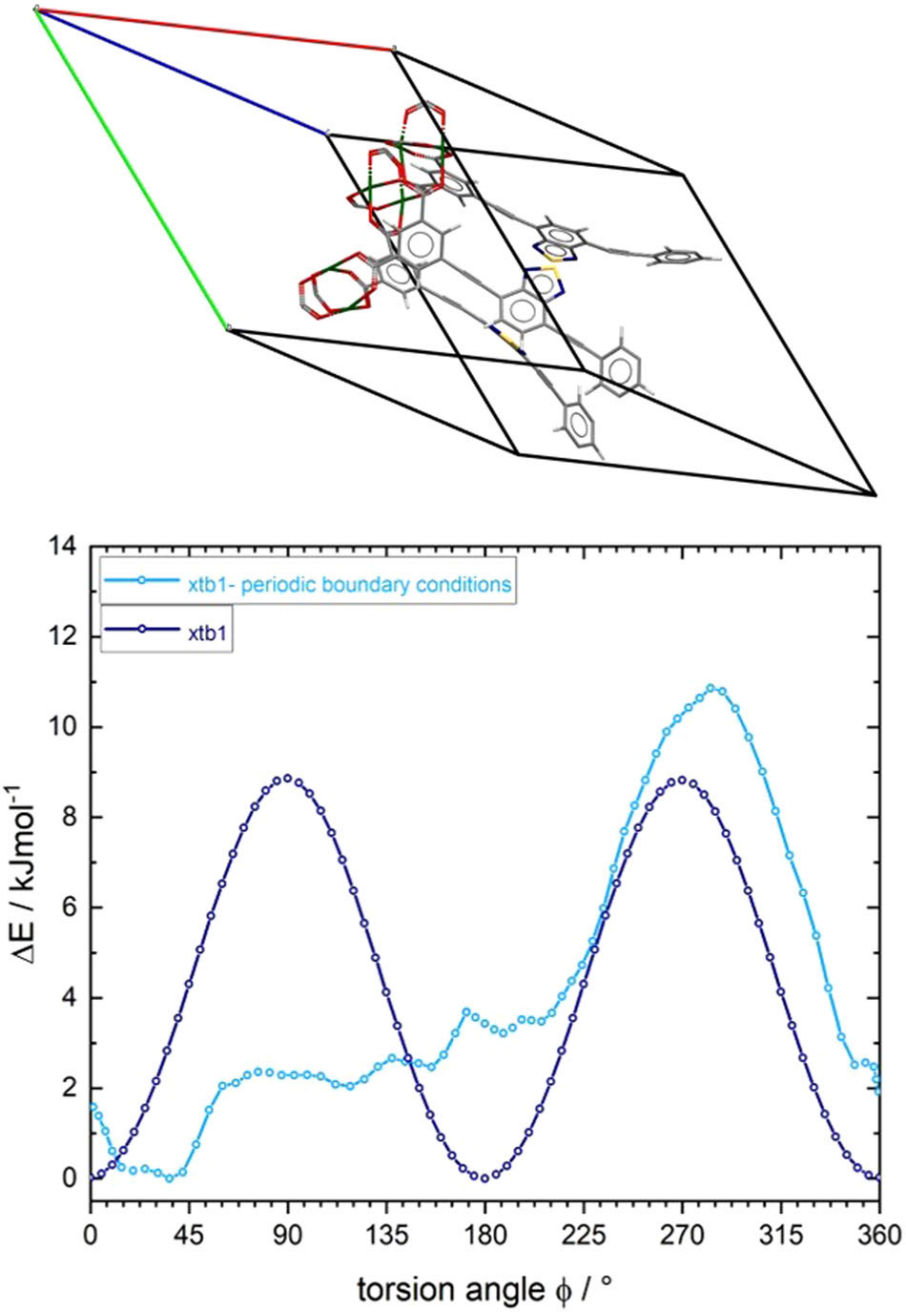
Comparison of calculated torsional potential curves considering 3D boundary conditions of JLU-Liu30. GFN-xtb1 calculated torsion potential curves for the rotation of a single benzothiadiazole rotor placed in cluster compound **2c** (cf. Fig. 8; dark-blue curve) and in the primitive unit cell of JLU-Liu30 (light blue curve), respectively.

Similar to the model compounds 1a-c, the size of the molecular fragment 2a-c has only a faint influence on the calculated torsion energy values, including the presence or absence of coordinated water molecules. All three methods predict different maximum potential energy barriers for the rotation of the rotor (ranging from 1.1 to 17 kJ/mol), essentially undergoing 180° flips with respect to its stator. GFN-FF completely underestimates the rotational barrier. This is however expected because the forcefield definition does not contain any force field term covering the torsion of fragments around a triple bond, (which is missing in all current force-fields to the best of our knowledge). The relatively high barrier of about 17 kJ/mol found in DFT calculations employing the r^2^SCAN-3c functional is rather surprising and demands a thorough check against other DFT functionals or higher levels of quantum mechanics. In relation to the experimentally determined rotational barrier from the DES-measurements of 17 kJ/mol, the calculated rotational barrier agrees well. Traceable to the dipole-dipole interaction within the SBU´s of both MOF systems, the 3D periodic fragments cannot be extended by the already listed calculation methods. To estimate the influence of intermolecular dipolar interactions in both frameworks, the torsion potentials of a single rotor under 3D periodic boundary conditions (Supporting Information) has been examined by climbing image nudged elastic band (CI-NEB) calculations. In Figs. 11 and 12, we compare the potential energy curves for the torsion of isolated rotors in a cluster fragment with the values obtained for rotors embedded within the crystal lattice. For ZJNU-40 we see only marginal differences for the potential energy curves gleaned from aperiodic and periodic models of the framework (Fig. 11). In contrast, the same calculations performed on JLU-Liu30 (Fig. 12) and its cluster model show a strong distortion of torsion potential for a full 360° rotation. The potential curve in the latter case becomes asymmetric, indicating a ratchet-type behavior. This behavior can be rationalized by the close-spaced circular arrangement of rotors in JLU-Liu30, where triples of rotors form a circular head-to-tail arrangement in the geometry optimized lattice structure. A similar arrangement is present in ZJNU-40. See Supplementary Data 1 and 2 for the corresponding trajectories.

The difference, however, is due to the high flexibility of the acetylenic linker in JLU-Liu30, which allows for a lattice distortion of the framework at which the rotors can approach each other at the closest possible distance, i.e., at van der Waals contact. This structural feature leads to stronger intermolecular coupling of the dipolar rotors in JLU-Liu30 as opposed to ZJNU-40.

In both compounds, the total change of activation energy needed for a full 360° linker rotation is small. This is presumably due to both, the low dipole moment of individual rotors on one hand and to the on average small contribution of dispersive rotor interactions on the other hand. In sum these effects lead to a slight (24%) increase of the torsion potential energy for JLU-Liu30. DFT calculations based on the r^2^SCAN-3c potential are expected to show similar trends. For visualisation purposes, movies showing the 360° rotation of the individual benzothiadiazole units of the two MOFs in the respective crystal lattices are included in the Supplementary Information (Supplementary Movie 1 and 2).

## Conclusions

Summarizing the dielectric-spectroscopy results, in both investigated MOFs we found clear evidence for relaxation dynamics, arising from the cooperative reorientational motions of the dipolar benzothiadiazole moieties in the linkers. It is characterized by non-exponentiality of the spectral shape and non-Arrhenius behavior of the relaxation time, typical for glassy freezing, and an orientational glass transition is approached upon cooling. Overall, the characteristics of the dipole dynamics in these systems, including the relatively moderate fragility, resembles the findings in other crystalline materials with reorientational degrees of freedom as the plastic crystals. From the temperature dependence of the relaxation times, the energy barriers for single-dipole rotation were deduced to be 27 kJ/mol and 17 kJ/mol for ZJNU-40 and JLU-Liu30, respectively. One should be aware, however, that the actual energy barriers, revealed by dielectric spectroscopy, are temperature dependent and enhanced due to cooperative dipole motions.

All our dielectric data on JLU-Liu30 are well consistent with an antiferroelectric ordering of the dipoles, located on the linkers of this MOF, below about 300 K. In recent years, there have been various reports of antiferroelectric ordering in MOFs, e.g., refs. ^13,71–77^. However, to our knowledge, only in a single case the ordering was found to arise from reorienting linkers^13^. Similar to order-disorder ferroelectrics, and just as in the antiferroelectric cyanides KCN and NaCN, below the polar phase transition dipoles that are not involved in the polar order still can reorient. Our finding of characteristic properties of glassy freezing for this dynamic reminds of the behavior in relaxor ferroelectrics^78,79^. If we assume a second-order antiferroelectric phase transition as in the cyanides, the order parameter should continuously increase below *T*_c_ and reach its maximum (i.e., full antiferroelectric order) for *T* → 0 K only. Interestingly, the orientational glass transition at 182 K for JLU-Liu30 will prevent this complete ordering unless for infinitely slow cooling rates.

Out of the torsion potential calculations employing a semi-empirical ansatz, namely GFN-xtb1, turn out to provide a balanced compromise between accuracy and costs (in terms of CPU hours) for estimating activation energy values for the rotation of dipolar rotors mounted between stators.

It should be mentioned here that all efforts to further increase the size of the model compounds had limited success, because the constrained torsion scans became numerically unstable. Trials of calculating molecular fragments comprising multiple rotors, for instance, indicated a strong influence of the calculated torsion potential parameters on the starting configurations. Calculations providing smooth torsion potentials would require the possibility for performing gradually iterative torsion scans based on a low-energy configuration resulting from a previous calculation step—a systematic scanning approach not yet implemented in the ORCA code (V.5.3). A theoretically determined rotational barrier of 24- 30 kJ/mol can be calculated for ZJNU-40 depending on the theoretical level. From the dielectric experiments, we were able to determine a rotational barrier for single dipoles of 27 kJ/mol, by the Vogel-Fulcher-Tammann approximation of the temperature-dependent relaxation time. For the GFN-FF method in particular, we found good agreement of the calculated rotational barriers of the individual molecular fragments with the experimentally determined data.

For the second system JLU-Liu30, depending on the theoretical level, a rotational barrier of 1.1–17 kJ/mol was calculated. Especially the calculation by the DFT method, r^2^SCAN-3c, yielded a rotational barrier of the rotor that very well compares to the experimentally determined by dielectric spectroscopy. The GFN-FF method as well as the GFN-xtb1 method, on the other hand, lead to much lower rotational barriers for the JLU-Liu30 system, which are at variance with the DES measurements.

In summary, in both MOFs, based on the experimentally determined data, rotational processes due to the benzothiadiazole moiety were detected and the resulting rotational barriers for single-dipole dynamics are consistent with the theoretically calculated values. Along with this, the identification of existing or the development of new dipolar MOF structures that exhibit distinct structural phase transitions with respect to the arrangement of the dipolar rotors supported by the framework structures seems to be an obvious goal for future research directions in this field. Here, the physical effects might become quite complex, similar to the many reported cases of magnetostructural phase transitions in ferro- or ferrimagnetic solid state compounds (vide infra).

Demonstrating activated diffusion, i.e. mass transport of adsorbed molecules as response to external stimuli yet represents another direction into which such framework compounds might be engineered, provided that appropriate design rules become available, which should put the required strong dipolar coupling of the rotor units into a balance with the requirements for mechanically and chemically stable frameworks. In terms of theoretical simulations, the inherent size-dependency for the emergence of permanently or switchable polarized domains remains a serious pitfall, demanding suitably optimized force-field approaches and a hierarchy of computational sampling and embedding techniques, as described and discussed by Goodwin^80^.

## Methods

### Experimental and computational details

All chemicals were purchased from commercial suppliers and were used in the condition received. The two linker synthesis of ZJNU-40 and JLU-Liu30 were synthesized via Suzuki-Miyaura reactions according to the literature procedures under reduced argon atmosphere^15,18^. The synthesis of the two MOFs ZJNU-40^14^ and JLU-Liu30^15^ were modified. The green crystals of ZJNU-40 were obtain by mixing Cu(NO_3_)_2_ ∙ 3H_2_O in Diethylformamide (DEF), adding the organic linker 5,5′-benzo[c][1,2,5]thiadiazole-4,7-diyldiisophthalic acid (H_4_L), water and 6 M hydrochloric acid. The mixture was heated in an oven at 70 °C over 96 h. JLU-Liu30 was prepared via autoclave synthesis at a temperature of 85 °C over a time of 48 h. For the likewise green crystals, Cu(NO_3_)_2_ ∙ 3H_2_O was dissolved in DEF. The linker 5,5′-benzo[c][1,2,5]thiadiazole-4,7-diylbis(ethyne-2,1-diyl)diisophthalic (H_4_btadpa), water and hydrochloric acid were appended to the mixture. The reference structure NOTT-101 was synthesized as stated of He et al.^16^. See Supplementary Methods for full details.

The materials were characterized by X-ray powder diffraction (XRPD), variable temperature X-ray diffraction, thermogravimetric analysis (TGA), Fourier-transform infrared (FTIR) spectroscopy, optically by scanning transmission electron microscope (STEM) and by an optical microscopy. The characterisations are described in Supplementary Data Figs. S1–S13 in the Supplementary Methods.

Full rotation torsion potentials were calculated for each of these fragments with the ORCA code (V.5.2 & V5.3)^81,82^.

Prior to the dielectric measurement, each sample was dried in vacuum atmosphere at 100 °C for 2 h in order to minimize the amount of residual solvent molecules and water in the sample. For JLU-Liu30, a dielectric measurement of the as-prepared material revealed an additional significant relaxation peak while the amplitude of the linker-related process remained essentially unchanged. Its absence in the dried sample demonstrates the effectiveness of the heat treatment. The powder samples were measured in a stainless-steal, circular, parallel-plate capacitor with a diameter of 10.0 mm and a plate distance of 0.17–0.40 mm, depending on the sample. The large ratio between capacitor plate area and capacitor plate distance minimizes stray-effects. All measurements were performed in a Quatro cryosystem by Novocontrol in a steady stream of dry N_2_-gas, employing a frequency-response Alpha-A analyzer by Novocontrol using pseudo-fourpoint contact geometry. All samples were investigated in a broad frequency range from 0.1 Hz to about 1 MHz between 180 and 375 K.

For more details on the experimental procedures, see the Supporting Information.

## Supplementary information

Peer Review File

Supporting Information

Description of Additional Supplementary Files

Supplementary Movie 1

Supplementary Movie 2

Supplementary Data 1

Supplementary Data 2

## Data Availability

The data that support the findings of this study are enclosed in the supplementary material (Supporting information, Supplementary Movie 1 and 2 and Supplementary Data 1 and 2) and are available from the corresponding author upon reasonable request.
