# Supplementary material for: Benzothiadiazole-based rotation and possible antipolar order in carboxylate-based metal-organic frameworks
Source: Commun Chem. 2023 Jul 29;6:161. doi: 10.1038/s42004-023-00959-6 (PMC10387106; doi:10.1038/s42004-023-00959-6)
Supplement: Supplementary file 2 — Supporting Information [file 42004_2023_959_MOESM2_ESM.pdf]

Supporting Information

Benzothiadiazole-based rotation and possible antipolar order in carboxylate-based metal-organic frameworks

Jennifer Schnabel, [1] Arthur Schulz[2], Peter Lunkenheimer[2] and Dirk Volkmer[1*]

[1.] Chair of Solid State and Materials Chemistry, University of Augsburg, Institute of Physics, Universitaetsstrasse 1, 86159 Augsburg, Germany.
[2]Experimental Physics V, Center for Electronic Correlations and Magnetism, University of Augsburg, Institute of Physics Universitaetsstrasse 1, 86159 Augsburg, Germany

[*]Email: dirk.volkmer@physik.uni-augsburg.de

1 SUPPLEMENTARY METHODS 2
2 PXRD ANALYSIS 3
3 FOURIER-TRANSFORM INFRARED (FTIR) SPECTROSCOPY 5
4 THERMAL STABILITY 6
5 OPTICAL ANALYSIS 10
6 COMPUTATIONAL DETAILS 11
SUPPLEMENTARY REFERENCES: 11

1 Supplementary Methods

Materials

Chemicals and reagents were purchased from suppliers and used as received. For the analysis of the MOFs, the following methods were utilized: X-ray powder diffraction (XRPD), variable temperature X-ray powder diffraction, Fourier-transform-infrared spectroscopy (FTIR), thermogravimetric analysis (TGA), scanning transmission electron microscopy (STEM), and optical microscopy.

Physical Measurements

The chemical composition of the organic linkers was verified with ^{1}H and ^{13}C-NMR spectra recorded on a Varian 400 NMR Spectrometer.

X-ray powder diffraction (PXRD) data were collected in the 4-50° 2θ range using a Seifert XRD 3003 TT-powder diffractometer with a Meteor 1D detector operating at room temperature using Cu Kα_1 radiation (λ= 1.54187). The Fourier-transform infrared (FTIR) spectra were recorded in the range of 400- 4000 cm^{-1} with a measurement period of 32 scans on a Bruker Equinox 55 FT-IR spectrometer.

Thermal stability was examined by variable temperature X-ray diffraction and thermogravimetric analysis (TGA). Variable temperature X-ray powder diffraction data were measured under nitrogen atmosphere (70ml per minute) with an Empyrean (PANalytical) diffractometer equipped with a Bragg–Brentano HD mirror, a PICcel3D 2x2 detector and a Cryo & humidity Chamber CHC plus+ (Anton Paar). The temperature program included a heating rate of 10 °C min^{-1} and then 10 min isothermal between the measurements. The (TGA) was performed with a Q500 analyzer from TA Instruments. It was measured in a temperature range from RT to 700 °C under nitrogen atmosphere at a heating grade of 10 K min^{-1}.

The optical analysis of the crystals was carried out with a scanning transmission electron microscope (STEM) and Olympus IX70 microscope equipped with a camera. The STEM micrographs were recorded on a ZEISS Crossbeam 550 operated at 30 kV.

Synthesis of 5,5-(2,1,3-benzothiadiazole-4,7-diyl)(di(benzene-1,3-dicarboxylic acid) for ZJNU-40:

The linker was synthesized according to the literature of Zhao et al..[1] Therefore, dimethyl-5-(pinacolboryl)isophthalate (5.34g, 16.32 mmol), 4,7-dibromobenzo[1, 2, 5]- thiadiazole (2.00 g, 6.8 mmol), K_2CO_3 (24g, 240 mmol) and 0.30 g (0.4 mmol) of the $Pd(PPH_3)_2Cl_2$ catalyst were mixed together and dissolved in 50 ml of dioxane. The mixture was stirred under reflux in an argon atmosphere for 72 h. After the reaction time the mixture was extracted with water and CH_2Cl_2 and the organic layers were combined and evaporated. The crude product was purified by column chromatography with a mixture of $CHCl_3$: MeOH 10:1. The dry product was hydrolyzed with 6M NaOH, filtered, acidified with conc. HCl and washed with plenty of water. This step was repeated a second time until a yellow/green solid is given.

(Yield: 13% (0.9838 g, 2.11 mmol)), ^{1}H NMR (DMSO-*d*6) δ (ppm): 13.50 (s, 4H), 8.80 (d, 4H), 8.55(t, 2H), 8.08 (s, 2H).

Synthesis of ZJNU-40: The MOF-synthesis is a modified synthesis prescription inquired by Song et al.[2] $Cu(NO_3)_2 \cdot 3\ H_2O$ (50 mg, 0.207 mmol) was dissolved in 3 ml of DEF in a screw-capped vial (20 ml). To this mixture the organic linker H_4L (10 mg, 0.0215 mmol), 160 µl of water and 100 µl of 6 M HCl were added. The vial was screwed and shortly placed in an ultrasonic bath. After heating at 70°C for 96 h in an oven, the product was centrifugated and washed with 2 ml each of DEF and water and then dried at air. For activation of the product, the green crystals were heated under vacuum to 100°C for 1 h. (Yield: 63 % based on H_4L)

Synthesis of 5,5-[2,1,3-benzothiadiazole-4,7-diyldi(ethyne-2,1-diyl)]di(benzene-1,3-diccarboxylic acid) for JLU-LIU-30: The linker synthesis was synthesized according to Luo et al..[3] Dimethyl-5-ethynylbenzene-1,3-dicarboxylate (1.39g, 6.25 mmol) was dissolved in 5 ml Triethylamine and 5 ml DMF with addition of 4,7-dibromobenzothiadiazole (0.88g, 2.99 mmol), $PdCl_2(PPh_3)_2$ (7.7 mg, 0.011 mmol) and CuI (4.38 mg, 0.023 mmol). The mixture was stirred under Argon atmosphere for 24 h at a temperature of 70 °C. After this reaction time, NaOH (1.36 g, 3.96 mmol), 15 ml MeOH and 15 ml of water were added and stirred again for 5 h at a temperature of 105 °C. In the next step, few drops of 12M HCl were added till the pH-value changed between 1-2. The hydrolysis process was repeated one more time with 5 g of NaOH, 30 ml MeOH and 30 ml of water overnight at a temperature of 65°C. The precipitated solid was filtered and washed with 12 M HCl and plenty of water. The brown solid was dried at air. (Yield: 77%, 1.19g, 2.45 mmol) ^{1}H NMR (DMSO-d_6): δ (ppm): 13.49 (s, 4H), 8.47 (t, 2H), 8.30(d, 4H), 8.04 (s, 2H), ^{13}C (DMSO-d_6): δ (ppm): 166.10, 154.01, 136.07, 133.57, 132.65, 130.76, 123.04, 116.25, 113.50, 95.32, 87.32

Synthesis of JLU-LIU-30: For the MOF- synthesis, $Cu(NO_3)_2 \cdot 3\ H_2O$ (50 mg, 0.207 mmol) was dissolved in 3 ml DEF, H_4btadpa (10 mg, 0.0195 mmol), 160 µl of water and 100 µl of 6 M HCl were added. The mixture was sealed in 20 ml autoclave and heated in an oven at 85 °C for 96 h. The green crystals were collected by filtration, washed two times with DEF and dried at air. (Yield: 59% based on H_4btadpa). For activation of the product, the green crystals were heated under vacuum to 100°C for 1 h.

Synthesis of NOTT-101[4]: For the synthesis of the isoreticular MOF NOTT-101, the commercially available linker Terphenyl- 3, 3, 5, 5 – tetracarboxylic acid (50 mg, 123 µmol) was dissolved in 7.5 ml DMF, 3.75 ml dioxane and 3.75 ml water in a 50 ml round bottom flask. $Cu(NO_3)_2 \cdot 3\ H_2O$ (104 mg, 429.9 µmol) and three drops of concentrated HCl were added and the mixture was stirred at 80 °C under reflux over a period of 3 days. After 72 h, the synthesis was cooled down to room temperature, centrifugated and washed with 5 ml each of DMF and Aceton. The turquoise powder dried briefly at air.
(Yield: 75.5 % according to the linker). For activation of the product, the green crystals were heated under vacuum to 100°C for 1 h.

2 PXRD Analysis

In the following section, the measured XPRD-diffraction of the synthesized MOFs are listed and compared with the calculated diffractograms. The X-ray crystallographic coordinates for literature known structures are deposited at the Cambridge Crystallographic Data Centre (CCDC). The respective

X-ray crystallographic coordinates are given below the powder pattern. These data can be obtained free of charge from The Cambridge Crystallographic Data Centre.

All measured diffractograms evidence good conformance.

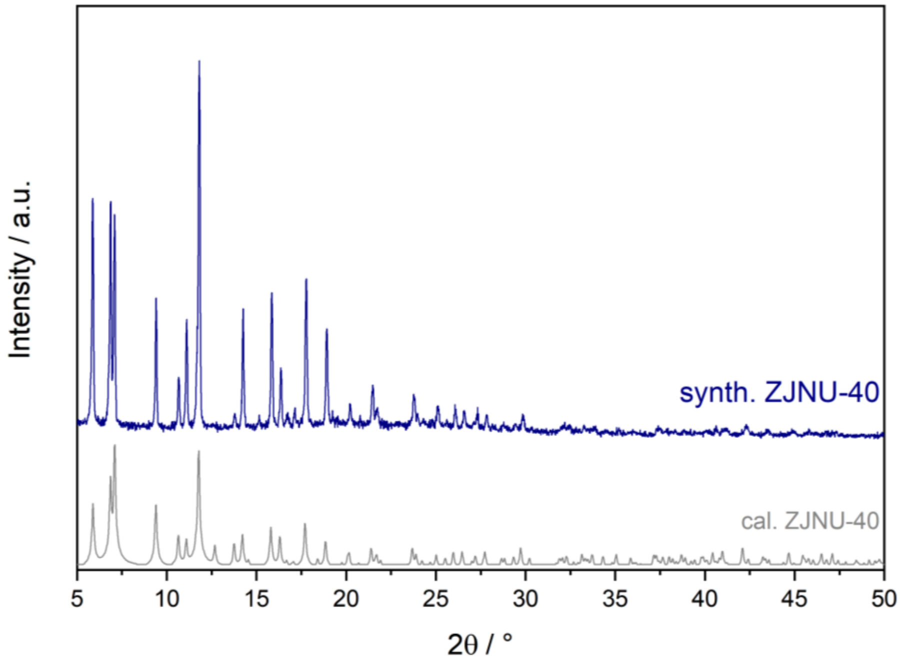

Figure S 1: PXRD of synthesized ZJNU-40 compared to the calculated MOF (CCDC: 1014276).

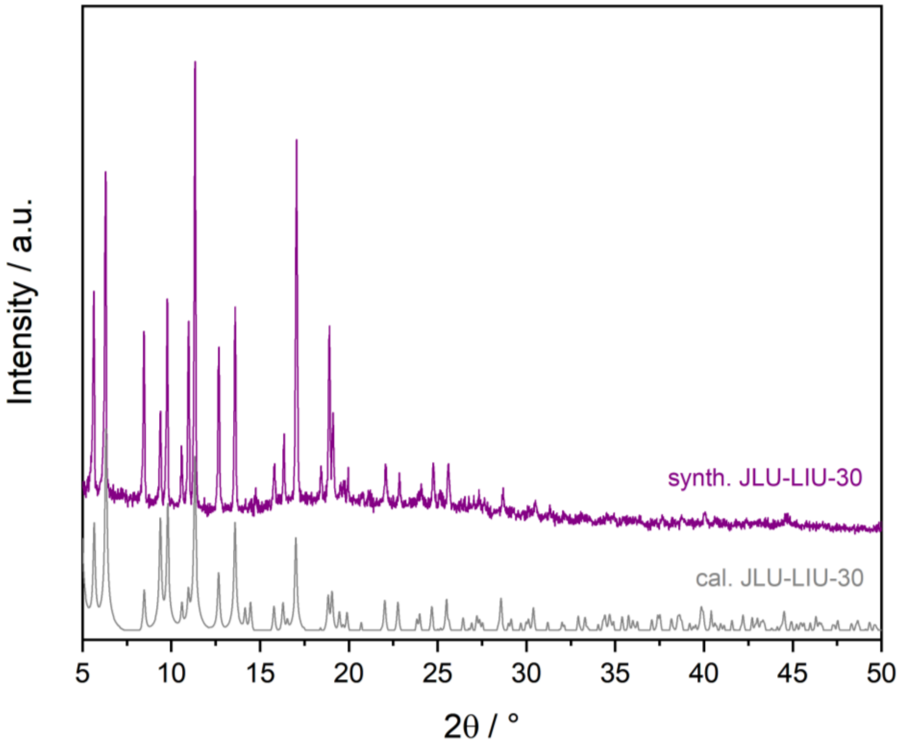

Figure S 2: PXRD of synthesized JLU-LIU-30 compared to the calculated MOF (CCDC: 1479874).

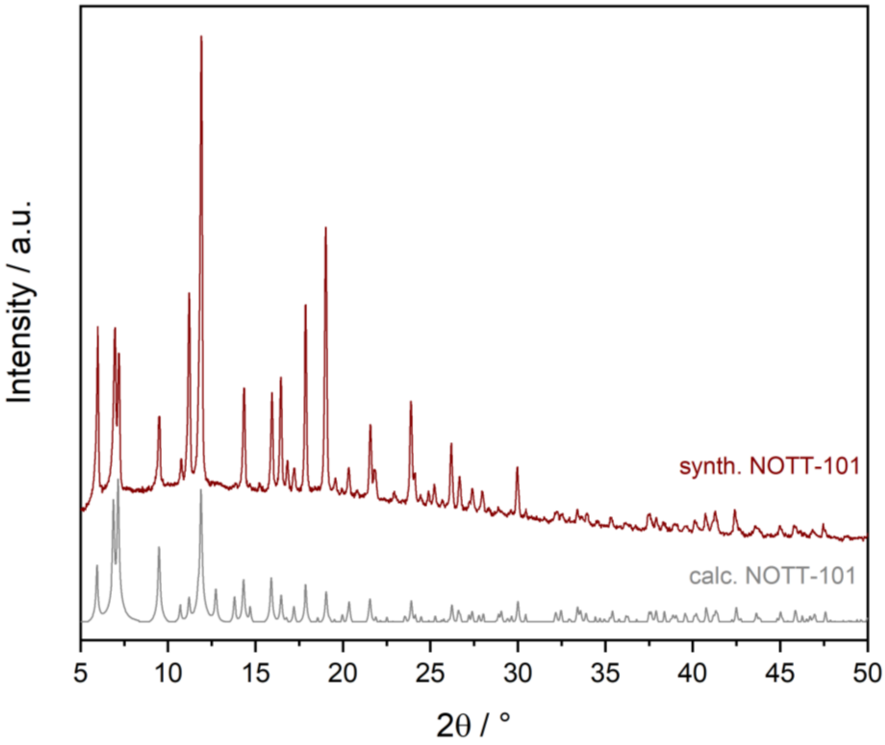

Figure S 3: PXRD of synthesized NOTT-101 compared to the calculated MOF (CCDC: 606908).

3 Fourier-transform infrared (FTIR) spectroscopy

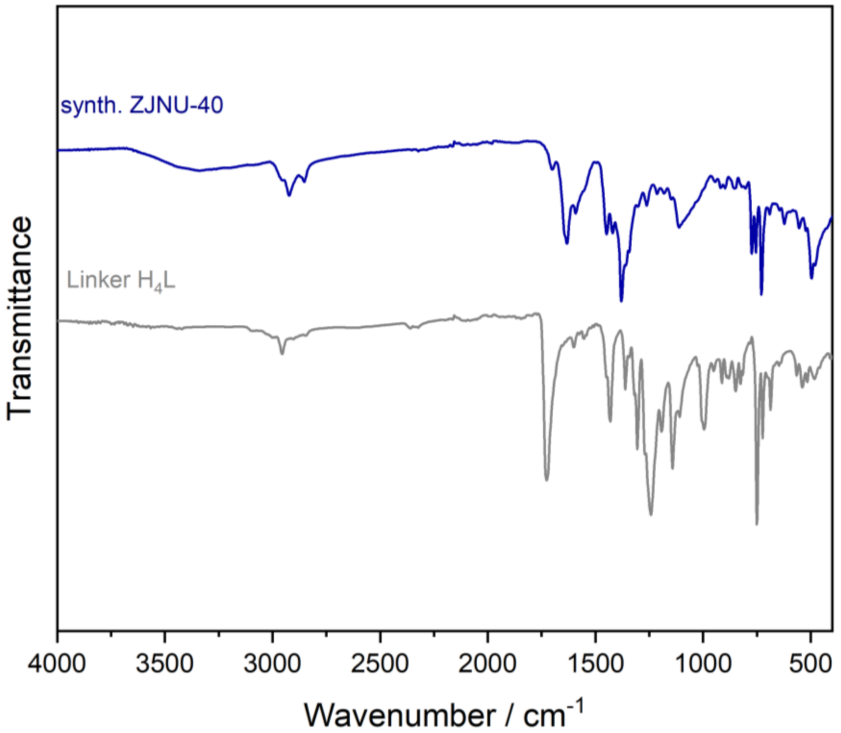

Figure S 4: FTIR-spectra of ZJNU-40 compared to its linker H_4L.

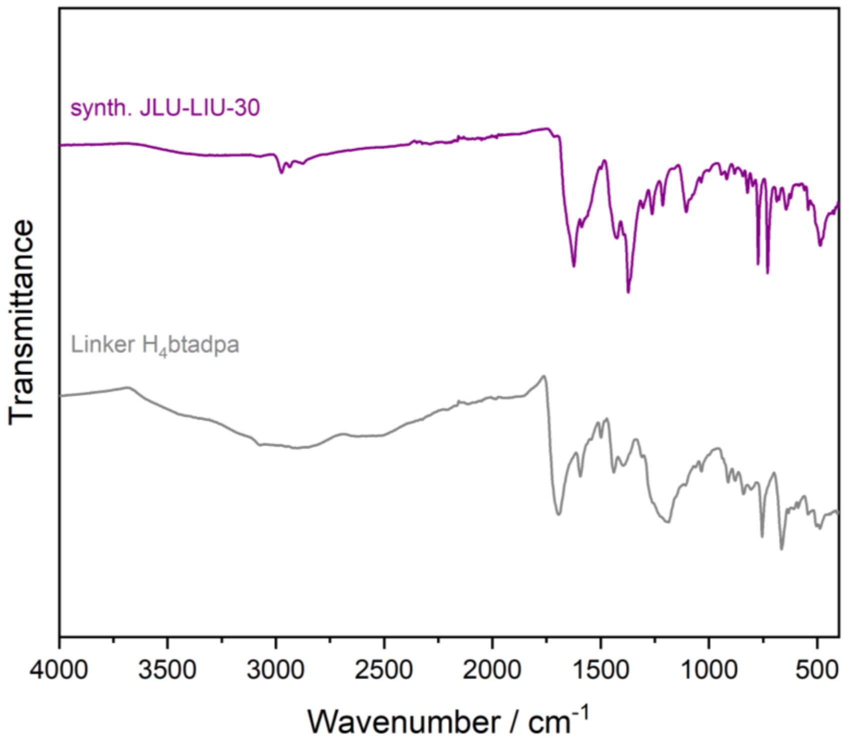

Figure S 5: FTIR-spectra of JLU-LIU-30 compared to its linker H_4btadpa.

4 Thermal Stability

In the following section, the thermal stability of ZJNU-40 and JLU-LIU-30 are explained in more detail. Based on these measurements, the activation protocol of the MOFs was adapted.

Thermal stability was examined by variable XPRD and TGA. ZJNU-40 is thermally stable up to 190°C. Above a temperature of 200°C, the network decomposes. This is also confirmed by the TGA. In the first step, 18.13% of remaining solvent evaporated.

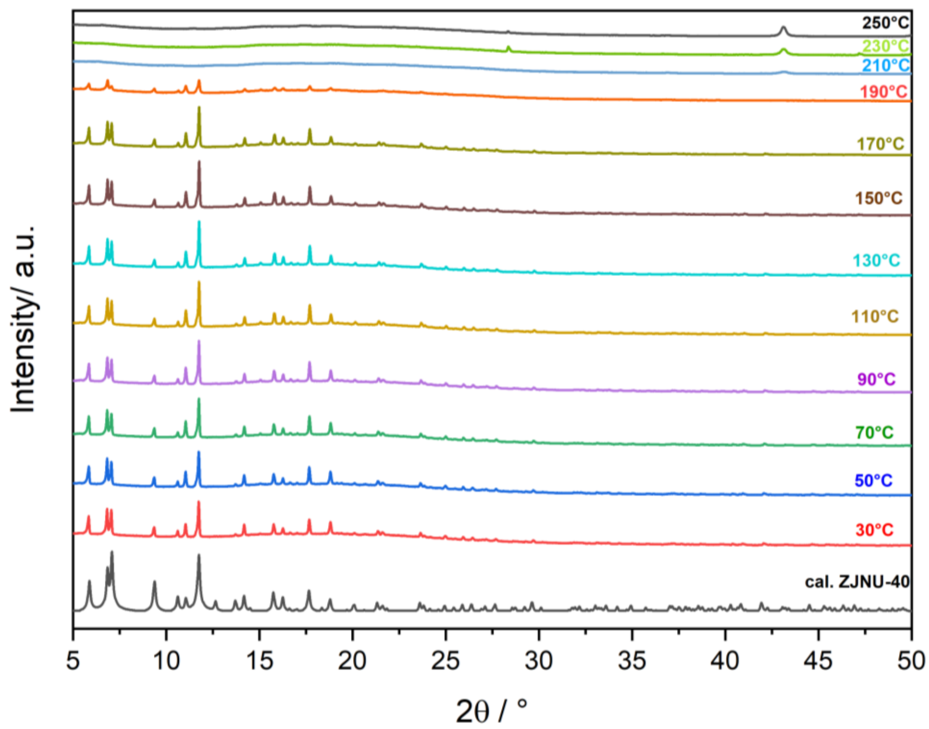

Figure S 6: Variable temperature-PXRD of synthesized ZJNU-40 compared to the calculated MOF at RT (CCDC: 1014276).

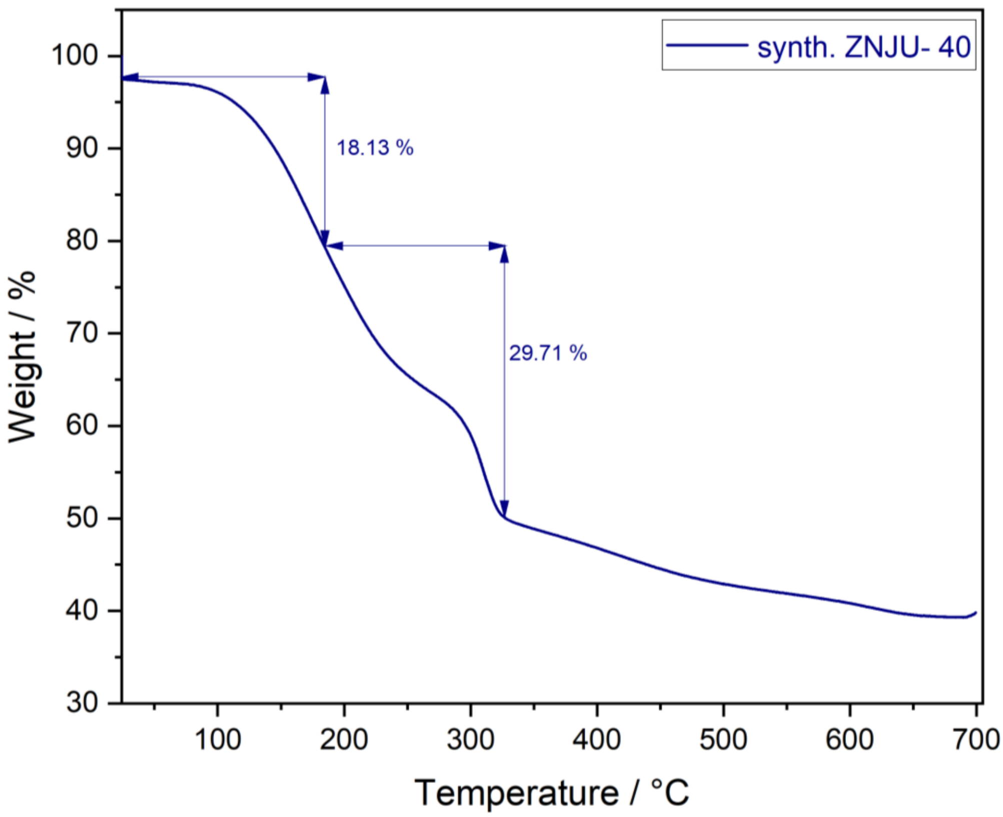

Figure S 7: TGA of synthesized ZJNU-40.

Crystallinity of JLU-LIU-30 remain up to a temperature of 120°C. The network collapse at a temperature up to 250°C.

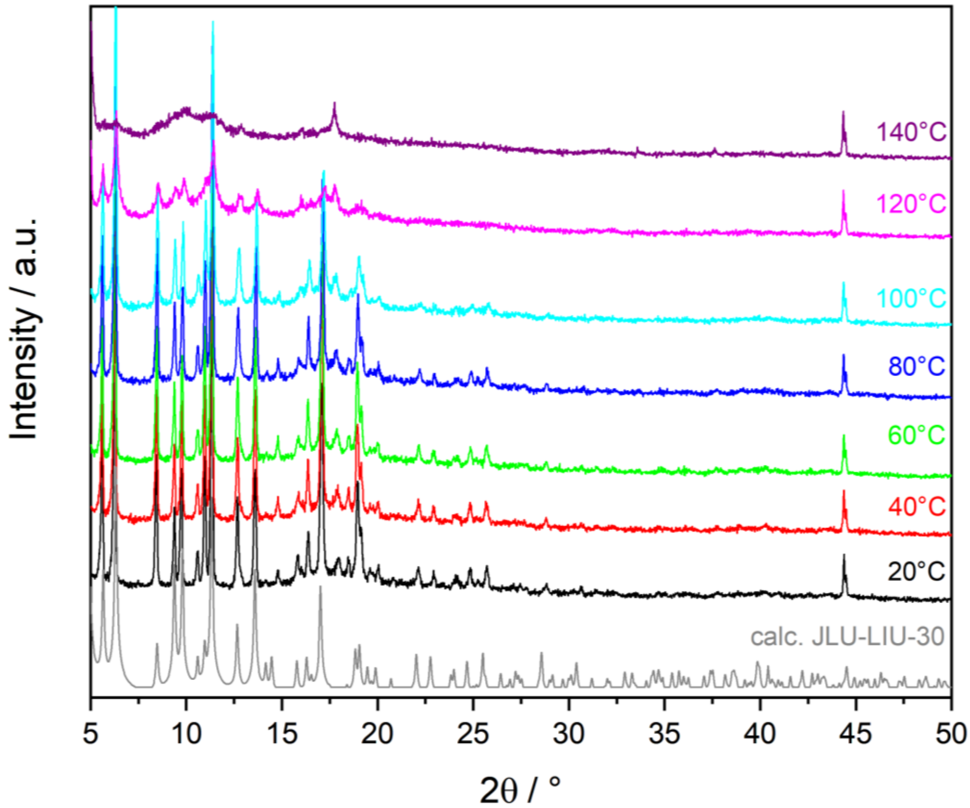

Figure S 8: Variable temperature-PXRD of synthesized JLU-LIU-30 compared to the calculated MOF at RT (CCDC: 1479874).

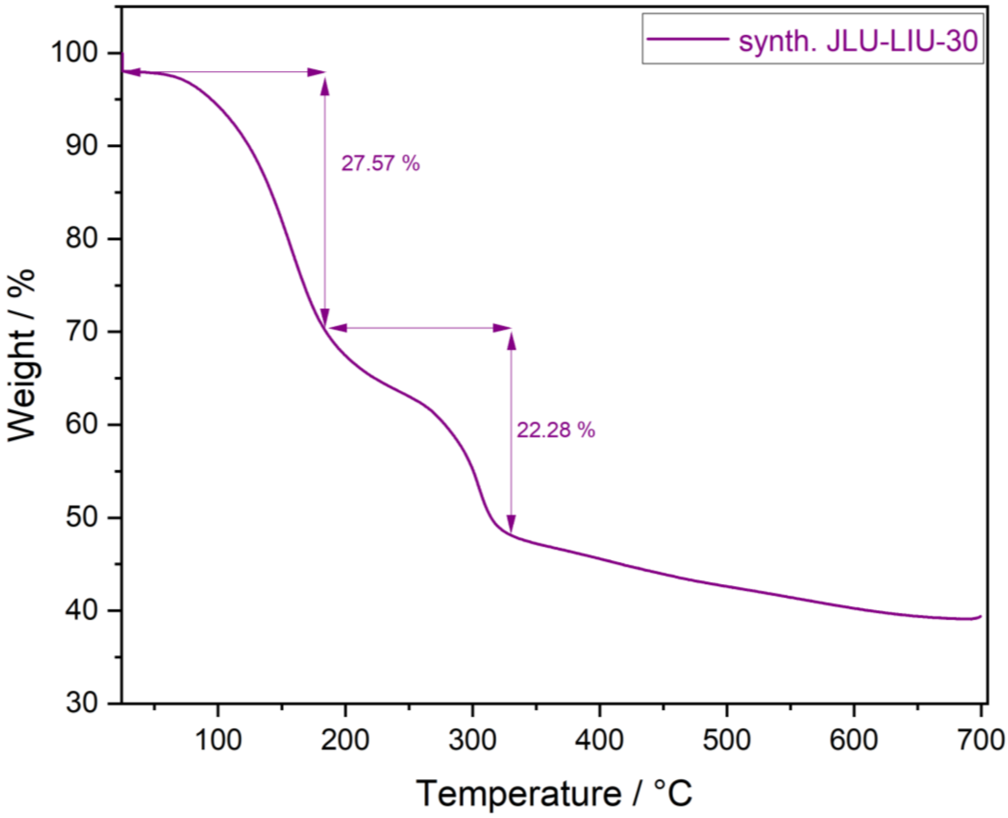

Figure S 9: TGA of synthesized JLU-LIU-30.

The isoreticular structure to both previous MOFs indicates a greater thermal stability. Characterized by variable temperature XRPD and TGA data, NOTT-101 is thermally stable up to 300°C until the network collapses. In the range between 100°C-150°C, excess solvent is removed from the structure.

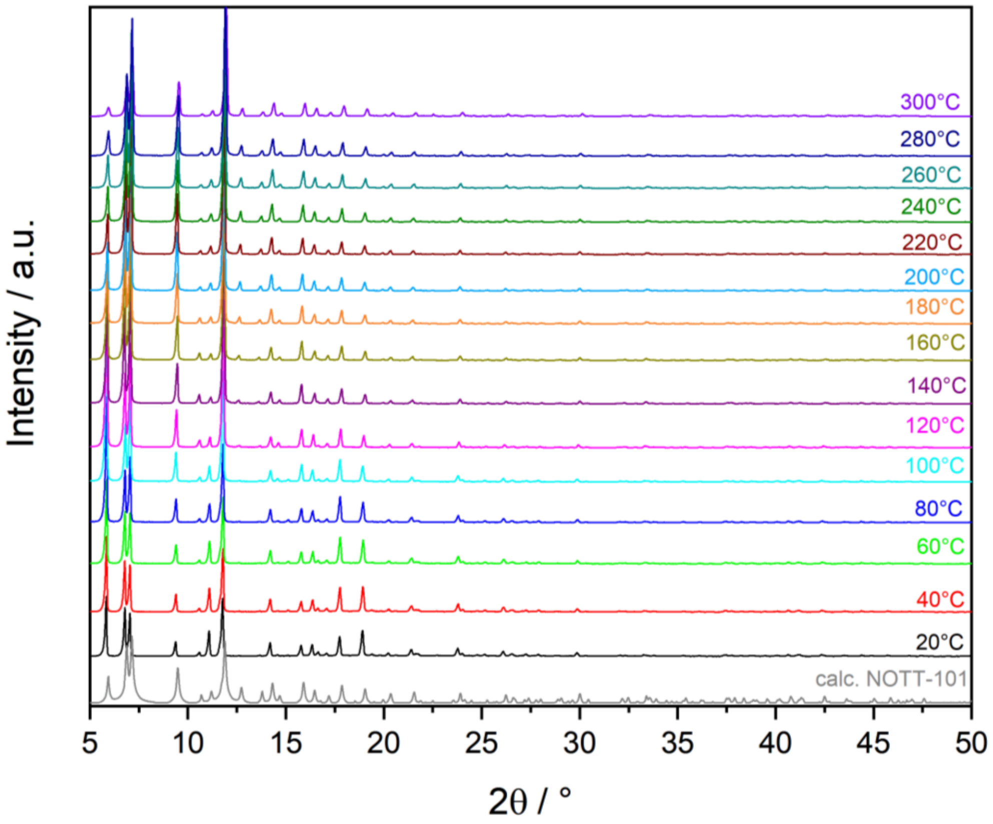

Figure S 10: Variable temperature-PXRD of synthesized NOTT-101 compared to the calculated MOF at RT (CCDC: 606908).

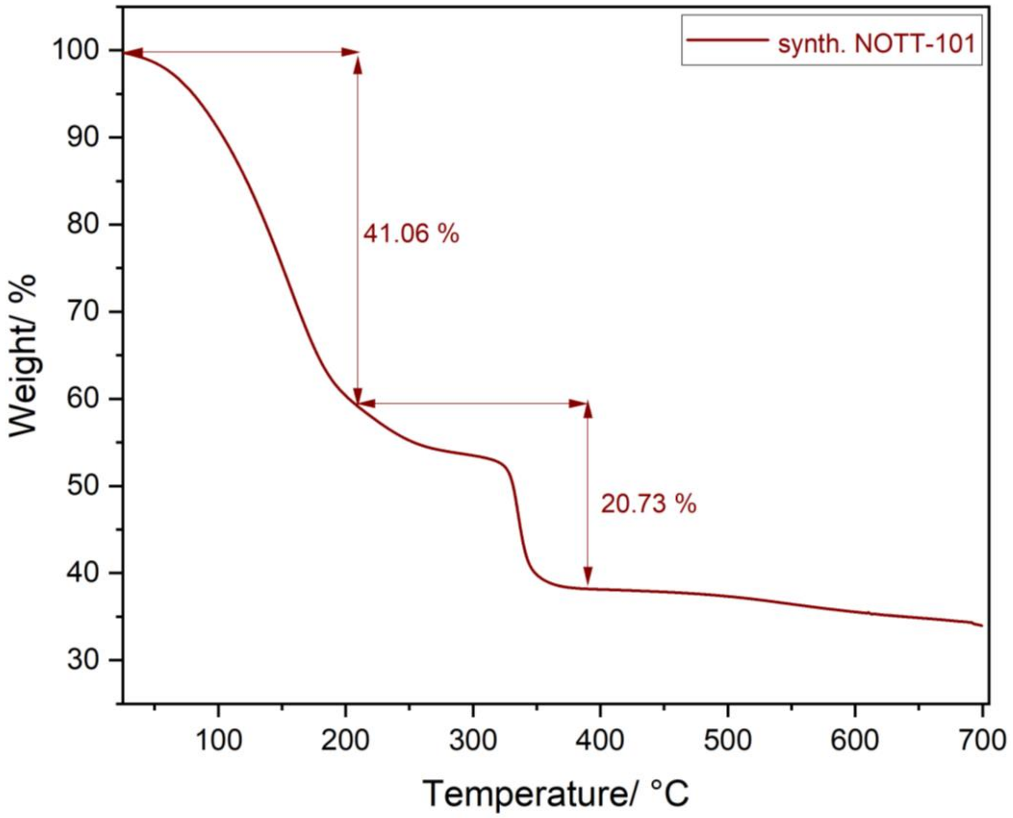

Figure S 11: TGA of synthesized NOTT-101.

5 Optical Analysis

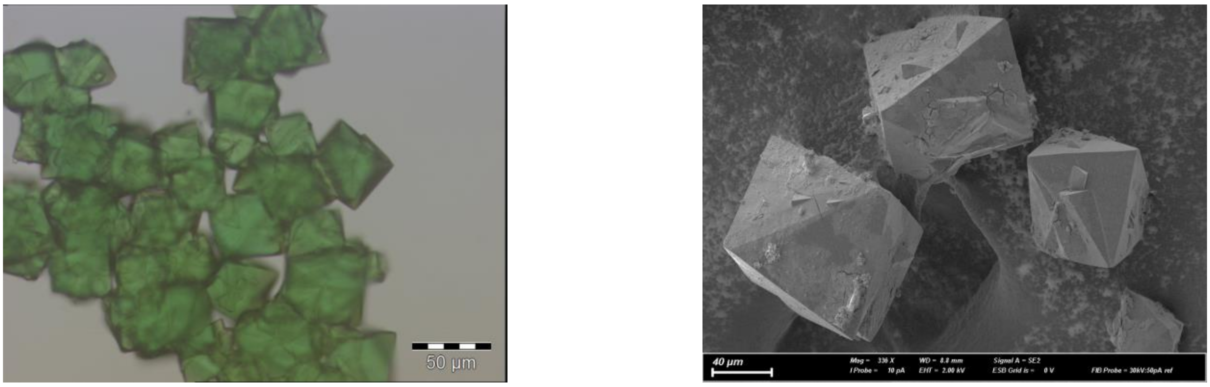

Figure S 12: left: recording of synthesized ZJNU-40 via optical microscope; right: recording of synthesized ZJNU-40 via STEM.

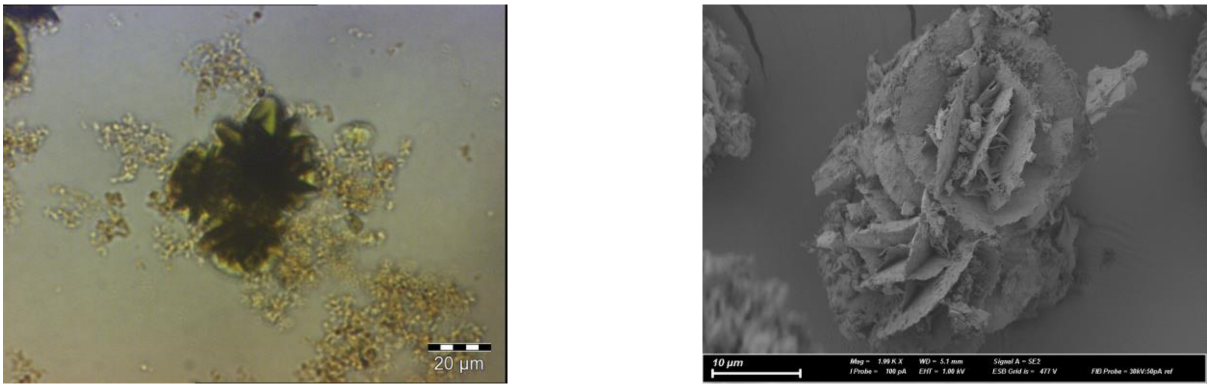

Figure S 13: left: recording of synthesized JLU-LIU-30 via optical microscope; right: recording of synthesized JLU-LIU-30 via STEM.

6 Computational details

Torsion potentials for molecular cluster models have been calculated with the ORCA program package, program version 5.0.3.[5] to which a compiled version of the xtb code (version 6.5.0) was dynamically linked.[6]

Calculations for the influence of intermolecular dipolar interactions of both framework structures on the torsional potentials under 3-D periodic boundary conditions were performed with the CP2k code, version 2023.17 using climbing image nudged elastic band (CI-NEB) calculations.

Owing to the typical slow convergence of such calculations and the huge number of periodic images which have to be calculated, we chose the GFN-xtb1 method for our calculations, which were performed on primitive reduced cells of the original hexagonal unit cells for both framework compounds. Each NEB contained 72 periodic images of the cell, that were constructed from 5 initial configurations in which the torsion angles of one of the btda rotors with respect to its isophthalic acid stators were set to values of 0, 90, 180, 270 and 360 deg., respectively. The starting torsion angles of the two other btda rotors were set at values corresponding to the fully relaxed lattice structures. The spring constant between the beads of the periodic images was set to 0.05, in conjunction with the following convergence criteria: max. force value 0.0010 and rms force value 0.0050.

Supplementary References:

1. Zhao, D. *et al.* Ratiometric dual-emitting MOF⊃dye thermometers with a tunable operating range and sensitivity. *J. Mater. Chem. C* **5**, 1607–1613 (2017).
2. Song, C. *et al.* Enhanced CO_2 sorption and selectivity by functionalization of a NbO-type metal–organic framework with polarized benzothiadiazole moieties. *Chem. Commun.* **50**, 12105–12108 (2014).
3. Luo, X. *et al.* Two Functional Porous Metal–Organic Frameworks Constructed from Expanded Tetracarboxylates for Gas Adsorption and Organosulfurs Removal. *Crystal Growth & Design* **16**, 7301–7307 (2016).
4. He, Y., Zhou, W., Yildirim, T. & Chen, B. A series of metal–organic frameworks with high methane uptake and an empirical equation for predicting methane storage capacity. *Energy Environ. Sci.* **6**, 2735 (2013).
5 Neese, F., Wennmohs, F., Becker, U. & Riplinger, C. The ORCA quantum chemistry program package. *The Journal of chemical physics* **152,** 224108, (2020).
6 Bannwarth, C. *et al.* Extended tight-binding quantum chemistry methods. *WIREs Comput Mol Sci* **11**; (2021).
7. Kühne, T. D. *et al.* CP2K: An electronic structure and molecular dynamics software package - Quickstep: Efficient and accurate electronic structure calculations. *The Journal of chemical physics* **152,** 194103; (2020).
