# Supplementary material for: Benzothiadiazole-based rotation and possible antipolar order in carboxylate-based metal-organic frameworks
Source: Commun Chem. 2023 Jul 29;6:161. doi: 10.1038/s42004-023-00959-6 (PMC10387106; doi:10.1038/s42004-023-00959-6)
Supplement: Supplementary file 3 — Description of Additional Supplementary Files [file 42004_2023_959_MOESM3_ESM.pdf]

Description of Additional Supplementary Files

File name: Supplementary Movie 1

Description: Simulated rotation of ZJNU-40

File name: Supplementary Movie 2

Description: Simulated rotation of JLU-LIU-30

File name: Supplementary Data 1

Description: ZJNU-40 trajectory

File name: Supplementary Data 2

Description: JLU-LIU-30 trajectory
