# Supplementary material for: Benzothiadiazole-based rotation and possible antipolar order in carboxylate-based metal-organic frameworks
Source: Commun Chem. 2023 Jul 29;6:161. doi: 10.1038/s42004-023-00959-6 (PMC10387106; doi:10.1038/s42004-023-00959-6)
Supplement: Supplementary file 1 — Peer Review File [file 42004_2023_959_MOESM1_ESM.pdf]

nature portfolio

Peer Review File

Benzothiadiazole-based rotation and possible antipolar order in carboxylate-based metal-organic frameworks

Open Access This file is licensed under a Creative Commons Attribution 4.0 International License, which permits use, sharing, adaptation, distribution and reproduction in any medium or format, as long as you give appropriate credit to the original author(s) and the source, provide a link to the Creative Commons license, and indicate if changes were made. In the cases where the authors are anonymous, such as is the case for the reports of anonymous peer reviewers, author attribution should be to 'Anonymous Referee' followed by a clear attribution to the source work. The images or other third party material in this file are included in the article's Creative Commons license, unless indicated otherwise in a credit line to the material. If material is not included in the article's Creative Commons license and your intended use is not permitted by statutory regulation or exceeds the permitted use, you will need to obtain permission directly from the copyright holder. To view a copy of this license, visit http://creativecommons.org/licenses/by/4.0/.

Reviewers' comments:

Reviewer #1 (Remarks to the Author):

In „Benzothiadiazole-based rotation and possible antipolar order in carboxylate-based metal-organic frameworks“ Schnabel et al. Investigate the field-induced dynamics of dipolar organic linkers incorporated in the backbone of two Cu(II)-based metal-organic frameworks (MOFs). The study is based on experimental and computational analysis on both MOFs for which the rotational/flipping barrier of the linker backbone is altered by different chemical modifications. The paper is well written and contains a large body of work. I particularly enjoyed the extensive comparison made to other materials in literature, in particular materials outside the MOF field. It is certainly of interest to a wide readership. I suggest publication in Communications Chemistry after the following points have been addressed.

P2 "any dipolar moments in its linkers" – I would rephrase this to no dipolar moments of freely movable linker parts" since there are dipole moments in this molecule but they are constrained due to lattice formation.

P3 "In the present case, however, we cannot fully exclude that reorientations of residual amounts of solvent molecules occluded in the pores of the MOF framework during sample synthesis could lead to the suggested secondary relaxation process, as was earlier found for MFU-4-type MOFs36."I think there is a word such as "which" missing in this sentence around synthesis

P3 "In the present case, we can only speculate about the nature of the charge carriers" – can defects in the lattice or partially reduced Cu be a source for this as well?

P6: "single-dipole
rotational energy barriers in ZJNU-40 and JLU-Liu-30, respectively" – it might be worth pointing out that this corresponds to a 180° fip and two of such barriers would need to be overcome for a full 360° rotation. Given that there is no directional information neither in the field nor the rotor it might be better to speak about a flip rather than a rotation. ◊ In the conclusion you refer to them as dynamics rather than rotation: "the energy barriers for single-dipole dynamics were deduced to be 27 kJ/mol and 17 kJ/mol for ZJNU-40 and JLU-Liu-30, respectively."

P6 "As shown in the inset," please assign this to a figure number

I particularily enjoyed the discussion about the "antiferroelectric polar ordering" and the extensive referencing of examples in other materials. Indeed I agree with the statement that the presence of minor mobile groups as in the MOFs might have a less significant role for a dipolar phase transition compared to the CN-examples listed in which the whole structure would also underog a change in structure and symmetry. DSC as a relatively sensitive method should still be able to detect any lare scale structural arrangement. For future work I suggest to give VT-SSNMR a try as this may also provide more details on the molecular nature of the dynamics.

Fig 8+9+10: For better comparison I suggest to directly compare dihedral scans of ZJNU-40 and JLU-LIU-

30 in a single plot and include these with an image of the respective fragment depicted in Fig 8 I suggest to move Fig9 and 10 to the ESI section.

P8 “potential energy barriers for the full rotation of the rotor with respect to its stator” would it be not more appropriate to assign this barrier for a 180° flip. Rotational barriers are often given for symmetric rotors but in this case a rotation clearly requires a two-step process.

General: There is quite a bit of discussion on the effects of solvents and other guest species on the Dielectric permittivity spectra. The authors state clearly that samples are to the best of their ability prepared under guest-free conditions. Can the effect be demonstrated by loading the powder and pores with a solvent and how would that impact the spectra? This could be a viable control experiment.

The method section of the manuscript is missing details of the experimental techniques, in particular the dielectric relaxation spectroscopy experiments. These can potentially be moved from the ESI.

Given the nice comprehensive study and extensive comparison of the observations with existing examples in literature I was a bit disappointed about the end of the paper. I think this nice piece of work deserves some perspective and outlook of which direction this research could move and which questions remain unanswered. Given that the dipolar ordering was found to only occur in a very few examples of "framework materials" the question arises if such phenomena would be present in other systems that have simply not been characterized.

ESI
The authors detail the thermal stability and describe the activation (solvent removal) procedure as: “heated under vacuum to 100°C for 1 h” – Given the high affinity of desolvated Cu-based MOFs towards moisture what measures were taken to prevent reabsorption of water during the physical measurements? In context to the DES measurements the authors state “in controlled N2-gas atmosphere”. How stable were the crystals under ambient conditions?

Reviewer #2 (Remarks to the Author):

With interest I read the paper on xxx by yyy et al. They employ broadband dielectric spectroscopy to unravel the dipolar dynamics of rotor linkers in two paddle wheel based metal-organic frameworks. The specifically probe the motion of the dipolar benzothiadiazole unit within two larger linkers, one where benzothiadiazole is connected to phenylene units, and one where it is connected to acetylene units. The expect faster dynamics (lower activation energy) in the latter case, which is also the motivation for their study. Finally they claim to find cooperative behaviour of the rotor-units, with glasslike freezing at very low temperatures, instead and for one of the MOFs a antiferroelectric phase transition at 300 K.
- Considering the motivation for their study, of rotor dynamics in MOFs with low energy barrier, and that they find cooperative motion of the linker, it is strange that the key papers on these two topics or not cited (or miscited). Regarding rotors (even dipolar rotors, with in some cases claims of antiferroelectric

order) the work of Angiolina Comotti should be explicitly cited and discussed: e.g. Angew. Chem. Int. Ed. 2023, 62, e202215893, JACS 2021, 143, 13082; Nature Chem. 2020, 12(9), 845. Similarly the pioneering work on rotors in MOFs with low activation barriers by Miguel Garcia-Garibay is missing, who also investigated dipolar rotors with claimed antiferroelectric ordering, e.g. Nature Chem. 2021, 13, 278 and PNAS, 2017, 114, 13613. The authors in their paper finally claim cooperative motion of the dipolar rotors based on the BDS data. This is not dissimilar from the paper on coupled rotor dynamics by a dipolar linker by Gonzalez-Nelson et al. JACS, 2021, 143, 12053. This paper is cited, however in the context of rotational motion related to gas absorption and storage. Which is not all what the paper is about. (typo: gases adsorb (not absorb) in MOFS). Very similarly to this work, the peaks in the dielectric loss spectra are significantly broadened compared to the relaxation of independent dipoles. Here the authors contribute it to cooperative dipolar interactions. In the paper by Gonzalez-Nelson it is contributed to steric interactions between neighbouring linkers, based on DFT and ab initio molecular dynamics calculations on supercells. The authors in the submitted work note that they could not do calculations with multiple linkers, because "Trials of calculating molecular fragments comprising multiple rotors, for instance, indicated a strong influence of the calculated torsion potential parameters on the starting configurations". This is precisely indicative of steric interactions between neighbouring linkers in their frameworks. The work should thus be discussed in this context.

- Regarding the conclusion of cooperative motion in the linkers due to dipolar interactions. There is no evidence given that the motion is cooperative, rather than just correlated or potentially coupled (cooperativity is a high bar, evoking images of gearlike motion, the motion is certainly restricted/influenced by the neighbours, but probably still dominated by thermal fluctuations). Neither is any evidence given for the statement that the interactions between the neighbouring molecules (which are evident from the BDS data) are dominated by dipolar interactions. Based on the current work, neither statement can be made. Moreover in all the body of work of observation of similar phenomena of glass like freezing by BDS certainly dipolar cooperative motion is not claimed as the only mechanism by which this is occurring. More precise insight in the phenomena observed by BDS (which provides hard to unambiguously assign data) is needed before acceptance of this paper. Certainly the calculations on multiple rotors are needed. The authors themselves indeed mention techniques on how to do this. It should be performed. Other linkers will then (probably) rotate out of the way, to obtain an energy minimized state for the linker on which a fixed torsional angle is applied. A precise discussion of the steric effects is needed. If the authors still wish to contrast this with potential dipolar interactions, a reliable estimate of the dipolar interaction energy needs to be made, and compared with thermal energy.
- The authors see for one the frameworks an anomaly in the dielectric spectra at 300K. The anomaly is clearly present. The authors find it more in accordance with antiferroelectric ordering than with ferroelectric ordering. That is also well-argues by the authors. But, like mentioned BDS provides hard to unambiguously assing data. While more in line with a phse transition involving antiferroelectric ordering than ferroelectric, that still doesn't mean it is antiferroelectric ordering. The authors should more critically discuss their claim of antiferroelectric ordering. What are potentially other phenomena that could give rise to such BDS spectra? And, what is the dipolar interaction energy compared to the thermal energy?
- A minor comment: the calculations of the torsional energy barriers based on force fields, and their comparison with ab initio calculations are interesting for people considering to use such force fields for similar calculations. However, only the ab initio data are sufficiently accurate to be of value for the

findings in the paper. Hence, I propose to move the FF-calculations to the SI.

Overall the work is very well reported, both the synthesis and the experimental techniques should allow others to reproduce the work.

Regarding whether the paper will influence the thinking in the field, will depend on how well the authors manage to shed light on the actual physical phenomena in these MOFs underlying their behaviour in BDS.

Reviewer #3 (Remarks to the Author):

The manuscript presents a detailed dielectric spectroscopic study of benzothiadiazole-based MOFs (ZJNU-40 and JLU-LIU-20) coupled with computational studies. The results indicate that at low temperatures and with an applied external field, the dipole moment of the benzothiadiazole results in the ordering of the ligands in the framework. The broad nature of the spectroscopic features is characteristic of glassy freezing. The Arrhenius behavior indicates cooperativity between units upon freezing. All of which is consistent with MOFs behaving like plastic crystals.

The observations of cooperativity complicate the application of computational studies (which do not account for interactions), but analysis of the higher temperature data presented is closer modeled by single rotor measurements. The barriers for rotation are calculated.

The discussion presented is comprehensive and the authors present clear arguments with appropriate references to explain the reported behavior.

The manuscript presents data to support a phenomenon that is predicted a priori through careful experimentation. The suitability for Comms Chem comes from the evaluation of novelty.

Response to Reviewers

Reviewer #1:

Reviewer comment:
In „Benzothiadiazole-based rotation and possible antipolar order in carboxylate-based metal-organic frameworks“ Schnabel et al. Investigate the field-induced dynamics of dipolar organic linkers incorporated in the backbone of two Cu(II)-based metal-organic frameworks (MOFs). The study is based on experimental and computational analysis on both MOFs for which the rotational/flipping barrier of the linker backbone is altered by different chemical modifications. The paper is well written and contains a large body of work. I particularly enjoyed the extensive comparison made to other materials in literature, in particular materials outside the MOF field. It is certainly of interest to a wide readership. I suggest publication in Communications Chemistry after the following points have been addressed.

Response:
We thank the reviewer for the careful reading of our manuscript and for his very positive comments. As detailed in the following, we made various revisions of the manuscript according to his suggestions.

Reviewer comment:
P2 "any dipolar moments in its linkers" – I would rephrase this to no dipolar moments of freely movable linker parts" since there are dipole moments in this molecule but they are constrained due to lattice formation.

Response:
We rephrased this sentence according to the reviewer's suggestion (page 2)

Reviewer comment:
P3 "In the present case, however, we cannot fully exclude that reorientations of residual amounts of solvent molecules occluded in the pores of the MOF framework during sample synthesis could lead to the suggested secondary relaxation process, as was earlier found for MFU-4-type MOFs36. "I think there is a word such as "which" missing in this sentence around synthesis.

Response:
We revised this sentence to make it clearer (page 3).

Reviewer comment:
P3 "In the present case, we can only speculate about the nature of the charge carriers" – can defects in the lattice or partially reduced Cu be a source for this as well?

Response:
In the revised manuscript (page 4), we now discuss some additional mechanisms that may generate charge carriers, leading to the observed small conductivity contribution: "Especially, Cu_2 paddle-wheel units were demonstrated to undergo partial decomposition by a variety of pathways. Todaro et al. for instance have investigated the slow hydrolysis reaction of Cu_2 paddle-wheel units in HKUST-1 at ambient conditions [Ref.: https://pubs.acs.org/doi/10.1021/acs.jpcc.6b03237] and they defined several models of defective paddle-wheel units by means of ESR spectroscopy. However, the time span during which slow decomposition was observed was about 20-50 days. Jeong et al. on the other hand demonstrated enhanced proton conductivity in HKUST-1, which was observed when methanol

was coordinated to the open metal sites of the Cu(II) ions in the paddle-wheel unit. [Ref.: https://pubs.acs.org/doi/10.1021/ja2110152]. A study conducted by Friedländer et al. [Ref.: https://pubs.acs.org/doi/10.1021/acs.jpcc.5b05019] showed that MOFs containing paddle-wheel units may contain a significant fraction of monomeric Cu(II) paddle wheel units. Hence, there are several well-documented cases that demonstrate a certain structural variability of Cu_2 paddle wheel units under different conditions. Moreover, electrically conductive surface species might yield an additional contribution to this effect in general. Given the fact that the observed conductivity is lower than $10^{-14}\ \Omega^{-1}cm^{-1}$ the estimated number of potential defect sites should be very small, which rules out the possibility to resolve the structural origin of this effect".

Reviewer comment:

P6: "single-dipole rotational energy barriers in ZJNU-40 and JLU-Liu-30, respectively" – it might be worth pointing out that this corresponds to a 180° flip and two of such barriers would need to be overcome for a full 360° rotation. Given that there is no directional information neither in the field nor the rotor it might be better to speak about a flip rather than a rotation. ◊ In the conclusion you refer to them as dynamics rather than rotation: "the energy barriers for single-dipole dynamics were deduced to be 27 kJ/mol and 17 kJ/mol for ZJNU-40 and JLU-Liu-30, respectively."

Response:

We added a sentence on page 2, explicitly stating that in fact the dipoles undergo flips between different angles, instead of a continuous rotation. We think this motion still may be called rotation, but we agree with the reviewer that its fliplike nature should be pointed out in the manuscript. We also rephrased the conclusion to avoid confusion,

Reviewer comment:

P6 "As shown in the inset," please assign this to a figure number

Response:

We added the figure number as suggested (Page 7).

Reviewer comment:

I particularily enjoyed the discussion about the "antiferroelectric polar ordering" and the extensive referencing of examples in other materials. Indeed I agree with the statement that the presence of minor mobile groups as in the MOFs might have a less significant role for a dipolar phase transition compared to the CN-examples listed in which the whole structure would also underog a change in structure and symmetry. DSC as a relatively sensitive method should still be able to detect any lare scale structural arrangement. For future work I suggest to give VT-SSNMR a try as this may also provide more details on the molecular nature of the dynamics.

Response:

We are glad that the reviewer enjoyed this section and thank him for his reasonable suggestion for future work.

Reviewer comment:

Fig 8+9+10: For better comparison I suggest to directly compare dihedral scans of ZJNU-40 and JLU-LIU-30 in a single plot and include these with an image of the respective fragment depicted in Fig 8 I suggest to move Fig9 and 10 to the ESI section.

Response:

Thank you very much for the suggestion. We have revised Figures 9-10 and added the Lewis formula to Figure 9-10 to make the assignment easier. In addition, we tried to implement the suggested changes and combine the graphs into a single plot. Unfortunately, this resulted in a confusing presentation. As we discuss the data in detail in the text, we would leave the graphs in the main script for better understanding.

Reviewer comment:

P8 "potential energy barriers for the full rotation of the rotor with respect to its stator" would it be not more appropriate to assign this barrier for a 180° flip. Rotational barriers are often given for symmetric rotors but in this case a rotation clearly requires a two-step process.

Response:

Whether the rotation process can be simplified to a 180° flip or not, depends on many aspects. From Figs 9 and 10 it becomes clear that the torsion potential for the benzothiadiazole rotor in ZJNU-40 shows twice the number of maxima and minima when compared to JLU-LIU-30. In both cases intrinsic electronic barriers play a most decisive role in defining the torsion-dependent energy values.

However, we extended the size of the rotor fragments cut out from the lattice in 3 steps, in order to show additional polarisation effects by the paddle-wheel units, for instance. In addition, we have now also added potential energy calculations for a 360° rotation of a single rotor in the framework under periodic boundary conditions (see below page 8 and page 10-11 in the manuscript), and these show clearly a symmetry breaking of the minimum energy path (MEP) a 360° rotation under the constraints of crystal symmetry. In the case of JLU-LIU-30 in particular, this leads to a non-symmetric potential, which requires a full 360° rotation of the torsion angle. Hence, diagrams 9 and 10 should retain the information of a full rotation, although (- we fully agree with the reviewer on this point-) the torsion angles ranging from 0-180° would contain sufficient information for the chosen molecular fragments.

Reviewer comment:

General: There is quite a bit of discussion on the effects of solvents and other guest species on the Dielectric permittivity spectra. The authors state clearly that samples are to the best of their ability prepared under guest-free conditions. Can the effect be demonstrated by loading the powder and pores with a solvent and how would that impact the spectra? This could be a viable control experiment.

Response:

We had earlier investigated in detail the effect of dipolar guest molecules like water or solvent in different MOF systems, see, e.g., ref. 36. We found that these molecules, as expected, lead to additional relaxation processes in the dielectric spectra. In all these experiments, we gained considerable experience about the proper procedures to remove such unwanted guest molecules. We are confident that the procedures applied to the present MOFs prior to the dielectric measurements, described in the Supporting Information, were fully sufficient to remove any notable amounts of unwanted dipolar molecules. However, we first measured an untreated sample to investigate the influence of residual solvent and indeed found an additional relaxation process, while the amplitude of the linker-related process remained essentially unchanged. We add this information to the experimental section on the dielectric measurements (now in the methods section, page 12-13).

We did not explicitly load large amounts of solvent into the pores to check additional influences.

Reviewer comment:
The method section of the manuscript is missing details of the experimental techniques, in particular the dielectric relaxation spectroscopy experiments. These can potentially be moved from the ESI.

Response:
We followed the reviewer's suggestion and have moved the description of the dielectric experiments to the methods section (page 12-13).

Reviewer comment:
Given the nice comprehensive study and extensive comparison of the observations with existing examples in literature I was a bit disappointed about the end of the paper. I think this nice piece of work deserves some perspective and outlook of which direction this research could move and which questions remain unanswered. Given that the dipolar ordering was found to only occur in a very few examples of "framework materials" the question arises if such phenomena would be present in other systems that have simply not been characterized.

Response:

We agree to the reviewer's statement that some perspective outlooks might be desirable. However, based on the behaviour shown by the two cases we have examined here we would like to refrain from adding strong advertising statements to the conclusion section which have no deep physical relation to the compounds we have investigated. However, we have added the following sentences (page 12)

"Along with this, the identification of existing or the development of new dipolar MOF structures that exhibit distinct structural phase transitions with respect to the arrangement of the dipolar rotors supported by the framework structures seems to be an obvious goal for future research directions in this field. Here, the physical effects might become quite complex, similar to the many reported cases of magnetostructural phase transitions in ferro- or ferrimagnetic solid state compounds (vide infra).

Demonstrating activated diffusion, i.e. mass transport of adsorbed molecules as response to external stimuli yet represents another direction into which such framework compounds might be engineered, provided that appropriate design rules become available, which should put the required strong dipolar coupling of the rotor units into a balance with the requirements for mechanically and chemically stable frameworks. In terms of theoretical simulations, the inherent size-dependency for the emergence of permanently or switchable polarized domains remains a serious pitfall, demanding suitably optimized force-field approaches and a hierarchy of computational sampling and embedding techniques, as described and discussed by A.L. Goodwin. [https://www.nature.com/articles/s41467-019-12422-z].

Reviewer comment:
ESI
The authors detail the thermal stability and describe the activation (solvent removal) procedure as: "heated under vacuum to 100°C for 1 h" – Given the high affinity of desolvated Cu-based MOFs towards moisture what measures were taken to prevent reabsorption of water during the physical measurements? In context to the DES measurements the authors state "in controlled N2-gas atmosphere". How stable were the crystals under ambient conditions?

Response:
The dielectric measurements were performed in a nitrogen gas cryostat, i.e., the sample was under a steady stream of dry nitrogen gas, excluding any water uptake. This was meant by "in controlled N_2-gas atmosphere" in the previous version and is now explicitly mentioned in the

revised methods section (page 13). Under ambient conditions (i.e., in air at room temperature), water uptake occurs; otherwise the sample is stable.

Reviewer #2:

Reviewer comment:
With interest I read the paper on xxx by yyy et al. They employ broadband dielectric spectroscopy to unravel the dipolar dynamics of rotor linkers in two paddle wheel based metal-organic frameworks. The specifically probe the motion of the dipolar benzothiadiazole unit within two larger linkers, one where benzothiadiazole is connected to phenylene units, and one where it is connected to acetylene units. The expect faster dynamics (lower activation energy) in the latter case, which is also the motivation for their study. Finally they claim to find cooperative behaviour of the rotor-units, with glasslike freezing at very low temperatures, instead and for one of the MOFs a antiferroelectric phase transition at 300 K.

Response:
We thank the reviewer for the careful reading of our manuscript and for his many detailed comments. We addressed all of them by making various revisions to the manuscript as detailed in the following point-by-point response:

Reviewer comment:
- Considering the motivation for their study, of rotor dynamics in MOFs with low energy barrier, and that they find cooperative motion of the linker, it is strange that the key papers on these two topics or not cited (or miscited). Regarding rotors (even dipolar rotors, with in some cases claims of antiferroelectric order) the work of Angiolina Comotti should be explicitly cited and discussed: e.g. Angew. Chem. Int. Ed. 2023, 62, e202215893, JACS 2021, 143, 13082; Nature Chem. 2020, 12(9), 845. Similarly the pioneering work on rotors in MOFs with low activation barriers by Miguel Garcia-Garibay is missing, who also investigated dipolar rotors with claimed antiferroelectric ordering, e.g. Nature Chem. 2021, 13, 278 and PNAS, 2017, 114, 13613. The authors in their paper finally claim cooperative motion of the dipolar rotors based on the BDS data. This is not dissimilar from the paper on coupled rotor dynamics by a dipolar linker by Gonzalez-Nelson et al. JACS, 2021, 143, 12053. This paper is cited, however in the context of rotational motion related to gas absorption and storage. Which is not all what the paper is about. (typo: gases adsorb (not absorb) in MOFS). Very similarly to this work, the peaks in the dielectric loss spectra are significantly broadened compared to the relaxation of independent dipoles. Here the authors contribute it to cooperative dipolar interactions. In the paper by Gonzalez-Nelson it is contributed to steric interactions between neighbouring linkers, based on DFT and ab initio molecular dynamics calculations on supercells. The authors in the submitted work note that they could not do calculations with multiple linkers, because "Trials of calculating molecular fragments comprising multiple rotors, for instance, indicated a strong influence of the calculated torsion potential parameters on the starting configurations". This is precisely indicative of steric interactions between neighbouring linkers in their frameworks. The work should thus be discussed in this context.

Response:
We have to agree with the reviewer on the inclusion of important key papers on rotor dynamics in MOFs. To better capture the motivation of the paper, the introduction has been rewritten (page 1). Thank you again for bringing this to our attention.

Furthermore, we want to point out that we do not claim that the detected broadening of the loss peaks evidences cooperative dipole motion. Any kind of interaction between the dipoles is sufficient to cause such broadening; it plays no role whether it causes cooperativity or not. Admittedly, this was not expressed sufficiently clear in the manuscript. In glass physics, it is the non-Arrhenius temperature dependence of the dipole dynamics, quantified by the

relaxation time, that is usually interpreted in terms of cooperativity of the dipolar motion (e.g., refs. 53-58). In contrast, the broadening of the relaxation peaks in the loss spectra nowadays is commonly ascribed to a distribution of relaxation times arising from the heterogeneity of the dipole dynamics (refs. 45, 46). Cooperativity is not necessary for this and such broadening does not prove cooperativity.

Heterogeneity means that each dipole has a somewhat different relaxation time because it senses a somewhat different environment, influencing its energy barrier for reorientation. This is obvious in amorphous materials like glasses or supercooled liquids, simply because of their structural disorder. However, in plastic crystals or other crystalline materials with dipolar reorientations, like the present MOF, such broadening is also commonly found, although they have a well-ordered crystalline lattice. There one can assume that the different environment of each dipole is caused by interactions with the neighboring dipoles whose orientations fluctuate and are disordered. They interact, e.g., via direct dipole-dipole interaction or steric hindrance, explaining the heterogeneity (ref. 35). For heterogeneity, causing the peak broadening, simply some kind of interaction between the disordered dipoles is necessary. It does not have to involve cooperativity, i.e., an interaction of dipoles in certain regions that leads to coupling of their dynamics.

We thank the reviewer for addressing this important point which was not discussed in sufficient detail in the manuscript. As a result, we now have made additions to the text explaining these issues comprehensively, also citing the work by Gonzalez-Nelson et al. *Nature communications* **10,** 4461 (2019) (page 4).

Reviewer comment:

- Regarding the conclusion of cooperative motion in the linkers due to dipolar interactions. There is no evidence given that the motion is cooperative, rather than just correlated or potentially coupled (cooperativity is a high bar, evoking images of gearlike motion, the motion is certainly restricted/influenced by the neighbours, but probably still dominated by thermal fluctuations). Neither is any evidence given for the statement that the interactions between the neighbouring molecules (which are evident from the BDS data) are dominated by dipolar interactions. Based on the current work, neither statement can be made. Moreover in all the body of work of observation of similar phenomena of glass like freezing by BDS certainly dipolar cooperative motion is not claimed as the only mechanism by which this is occurring. More precise insight in the phenomena observed by BDS (which provides hard to unambiguously assign data) is needed before acceptance of this paper.

Response:

The reviewer is right, from the present dielectric experiments alone, one cannot directly prove cooperativity in this material. However, in Fig. 5 we provide clear evidence for non-Arrhenius behavior of the dipolar dynamics. As mentioned above, in glass physics it is nowadays commonly accepted that non-Arrhenius behavior is due to cooperativity (see, e.g., refs. 53-58). This was experimentally proven, e.g., by nonlinear dielectric measurements performed by some of the present authors as published in Phys. Rev. Lett. **111**, 225702 (2013) and in ref. 51 (there the cooperativity is termed "dynamical correlation"). This is also valid for plastic crystals, see, e.g., our works in Phys. Rev. Lett. **114**, 067601 (2015) and J. Chem. Phys. **144**, 114506 (2016).

We think cooperative motion not necessarily has to be "gear-like" and other interactions may also lead to cooperativity as well. Here, we use the term "cooperativity" in the sense of the Adam-Gibbs theory of the glass transition [G. Adam and J.H. Gibbs, J. Chem. Phys. **43**, 139 (1965)] and of newer theories expanding it (e.g., Bouchaud&Biroli, Phys. Rev. B **72**, 064204 (2005). It means that the molecules "collectively rearrange over some length scale" (ref. 56) without specifying the type of interaction. While we do not have a direct prove that the non-Arrhenius behavior detected in these two MOFs is due to cooperativity, based on the current understanding of glassy freezing, it seems the best explanation. We have revised the manuscript text on page 5 and added some refences (55-58) to make this clearer.

The reviewer states that there is no evidence that the interactions between neighbouring molecules are dominated by dipolar interactions. This is true and here we have to admit that the phrasing in the text concerning interdipole interactions was often ambiguous. By expressions like "interactions between the dipoles", etc., we clearly did not want to say that these interactions are of direct dipole-dipole nature. The latter may play a role but there are many other possibilities how the dipoles can interact, e.g., via lattice strains or steric effects. We have now revised the corresponding passages to clarify this (pages 4 and 6). To further elucidate the interdipole interactions, we also performed additional calculations for rotors embedded within the crystal lattice as explained in detail below.

The reviewer remarks that BDS "provides hard to unambiguously assign data". Probably, he refers here to the possibility that residual amounts of solvent or water could cause the observed main dipolar relaxation process in the two materials. To check this, we have measured the isoreticular NOTT-101 MOF which lacks any dipolar linkers. We think that the absence of any significant relaxation process in the dielectric spectra of this reference system makes it very likely that the detected dipole motions are indeed due to the dipolar linkers.

Reviewer comment:

Certainly, the calculations on multiple rotors are needed. The authors themselves indeed mention techniques on how to do this. It should be performed. Other linkers will then (probably) rotate out of the way, to obtain an energy minimized state for the linker on which a fixed torsional angle is applied. A precise discussion of the steric effects is needed. If the authors still wish to contrast this with potential dipolar interactions, a reliable estimate of the dipolar interaction energy needs to be made, and compared with thermal energy

Response:

In order to determine more precisely the influence of the intermolecular dipolar interactions on the torsional potentials in both systems, we have computed the torsional potentials under 3D periodic boundary conditions and included them in our manuscript (page 10-11) and Supporting Information (section Calculation details). The torsional potentials were calculated by Climbing Image Nudged Elastic Band (CI-NEB) calculations and due to the typically slow convergence of such calculations and the large number of periodic images, we chose the GFN-xtb1 method for our calculations performed on the primitive reduced cells of the original hexagonal unit cells.
Two movies were also being made available which show the 360° rotation of single benzothiadiazole units in both lattices.

We have added the following sentences to our manuscript:

“To estimate the influence of intermolecular dipolar interactions in both frameworks, the torsion potentials of a single rotor under 3D periodic boundary conditions (Supporting Information) has been examined by climbing image nudged elastic band (CI-NEB) calculations.

In Figs. 11 and 12 we compare the potential energy curves for the torsion of isolated rotors in a cluster fragment with the values obtained for rotors embedded within the crystal lattice. For ZJNU-40 we see only marginal differences for the potential energy curves gleaned from aperiodic and periodic models of the framework (Fig. 11). In contrast, the same calculations performed on JLU-LIU-30 (Fig.12) and its cluster model show a strong distortion of torsion potential for a full 360° rotation. The potential curve in the latter case gets asymmetric, indicating a ratchet-type behavior. This behavior can be rationalized by the close-spaced circular arrangement of rotors in JLU-LIU-30, where triples of rotors form a circular head-to-tail arrangement in the geometry optimized lattice structure. A similar arrangement is present in ZJNU-40.

The difference, however, is due to the high flexibility of the acetylenic linker in JLU-LIU-30, which allows for a lattice distortion of the framework at which the rotors can approach each other at the closest possible distance, i.e. at van der Waals contact. This structural feature

leads to stronger intermolecular coupling of the dipolar rotors in JLU-LIU-30 as opposed to ZJNU-40.

In both compounds the total change of activation energy needed for a full 360° linker rotation is small. This is presumably due to both, the low dipole moment of individual rotors on one hand and to the on average small contribution of dispersive rotor interactions on the other hand. In sum these effects lead to a slight 24% increase of the torsion potential energy for JLU-LIU-30. DFT calculations based on the r^{2}SCAN-3c potential are expected to show similar trends."

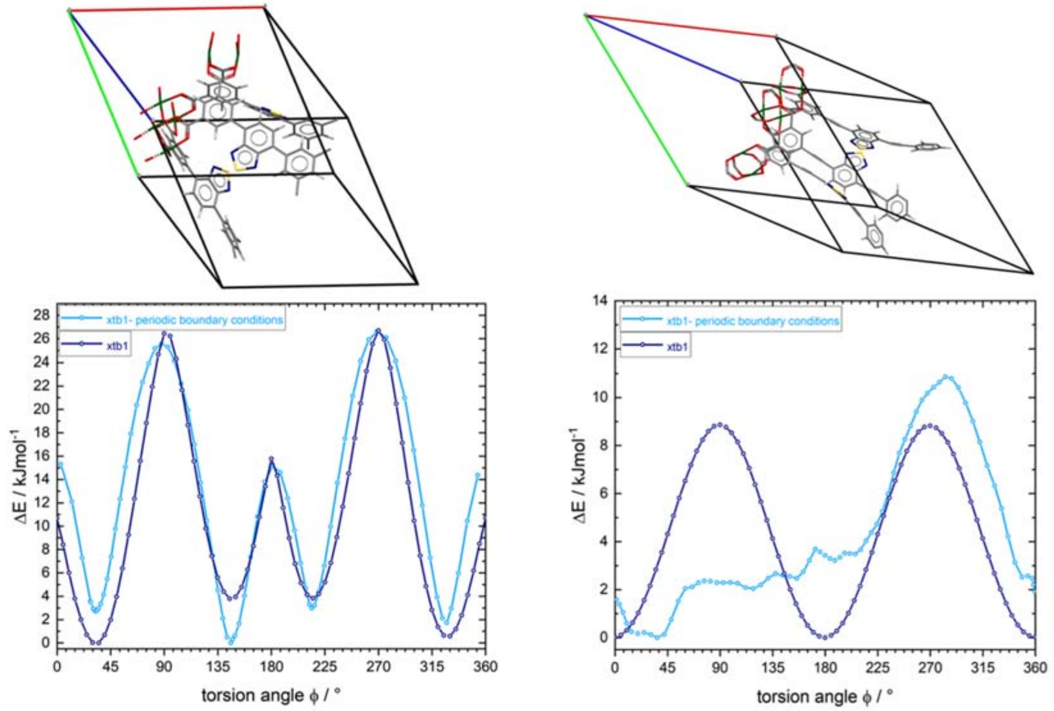

Reviewer comment:

The authors see for one the frameworks an anomaly in the dielectric spectra at 300K. The anomaly is clearly present. The authors find it more in accordance with antiferroelectric ordering than with ferroelectric ordering. That is also well-argues by the authors. But, like mentioned BDS provides hard to unambiguously assing data. While more in line with a phase transition involving antiferroelectric ordering than ferroelectric, that still doesn't mean it is antiferroelectric ordering. The authors should more critically discuss their claim of antiferroelectric ordering. What are potentially other phenomena that could give rise to such BDS spectra? And, what is the dipolar interaction energy compared to the thermal energy?

Response:

It is true that our experimental data cannot provide an absolute proof for antiferroelectric ordering, but we think that there are at least some strong hints. We want to remark that in fact we did not claim such a proof: For example, in the title "possible antipolar order" is mentioned, in the abstract we speak of "hints at antipolar ordering" and in the Conclusion section, we state that our dielectric data are "well consistent with an antiferroelectric ordering". The latter for sure is true, because: (i) we see a typical antiferroelectric anomaly in the temperature dependence, which should be no experimental artefact because it occurs upon cooling and heating, (ii) we detect relaxational dynamics above and below the suggested transition whose amplitude becomes reduced upon cooling, as expected for a second-order antiferroelectric transition, (iii) the dielectric behaviour closely resembles that in other antiferroelectrics as the cyanides.

Nevertheless, we now explicitly mention that our data do not provide a final proof of antiferroelectricity (page 7). If the material does not become antiferroelectric, the observed anomaly must be due to a different type of phase transition of unknown nature. We also have no alternative explanation for the anomalous temperature dependence of the relaxation strength below the transition.

Reviewer comment:
A minor comment: the calculations of the torsional energy barriers based on force fields, and their comparison with ab initio calculations are interesting for people considering to use such force fields for similar calculations. However, only the ab initio data are sufficiently accurate to be of value for the findings in the paper. Hence, I propose to move the FF-calculations to the SI.

Response:
We thank the reviewer for this suggestion. However, we disagree on the point of view that force fields are unsuitable for performing such calculations in general. In fact, the GFN-FF force field chosen for our calculations is a novel, partially polarizable generic force-field which should be able to cover crucial aspects of the static and dynamic polarisation of the framework compounds, at least from a technical point of view. Furthermore, we think that the direct comparison of the 3 different theoretical approaches (i.e., FF, tight-binding, and DFT) will provide some valuable hints to the readership for deciding which theoretical approach to employ when performing their own calculations.

Therefore, we have decided to leave the FF torsion potential curves in the main manuscript

Reviewer comment:
Overall the work is very well reported, both the synthesis and the experimental techniques should allow others to reproduce the work.

Response:
Thank you for this positive comment!

Reviewer comment:
Regarding whether the paper will influence the thinking in the field, will depend on how well the authors manage to shed light on the actual physical phenomena in these MOFs underlying their behaviour in BDS.

Response:
Concerning the interpretation of the dielectric data in terms of physical phenomena, we point to our responses listed above. Overall, we provide detailed and interesting experimental data (none of the referees doubts their reliability) and offer –as we think– reasonable and likely explanations for them. We think this is the normal procedure in the publication of experimental data. It certainly would be nice to have absolute final proofs for the correctness of the proposed interpretations but this is rarely the case in experimental work.

Reviewer #3:

Reviewer comment:
The manuscript presents a detailed dielectric spectroscopic study of benzothiadiazole-based MOFs (ZJNU-40 and JLU-LIU-30) coupled with computational studies. The results indicate that at low temperatures and with an applied external field, the dipole moment of the benzothiadiazole results in the ordering of the ligands in the framework. The broad nature of

the spectroscopic features is characteristic of glassy freezing. The Arrhenius behavior indicates cooperativity between units upon freezing. All of which is consistent with MOFs behaving like plastic crystals.

The observations of cooperativity complicate the application of computational studies (which do not account for interactions), but analysis of the higher temperature data presented is closer modeled by single rotor measurements. The barriers for rotation are calculated.

The discussion presented is comprehensive and the authors present clear arguments with appropriate references to explain the reported behavior.

The manuscript presents data to support a phenomenon that is predicted a priori through careful experimentation. The suitability for Comms Chem comes from the evaluation of novelty.

Response:

We thank the reviewer for the very positive review of our work!

REVIEWERS' COMMENTS:

Reviewer #1 (Remarks to the Author):

The authors have extensively addressed all questions raised by the reviewer and made substantial changes to the initial manuscript. Additional references were added to better provide a comprehensive overview of previous work. It becomes clear to me that much more in depth studies like the one in question need to be conducted to elucidate the effects observed, in particular with respect to aspects of collective/cooperative behavior. On the other hand a multi-experimental approach seems to be mandatory to further elucidate the mechanism and it will be a pleasure to observe this field emerge further. In the scope of the present work I feel that all relevant aspects have been addressed by the authors and think the manuscript can be published in the revised form.

In the light of the revisions provided I am also able to comment on the revisions made with respect to comments raised by reviewer #2 which I consider very valuable. The authors have added many relevant references at appropriate positions and thus also provided a much broader introduction into the topic. The authors have also addressed and rephrased discussions on aspects of cooperative dipole motion which is not indefinitely supported by the experimental and computational data. I feel that the now much deeper discussion on cooperativity and the different meanings associated to this property (gear-like vs collective) makes this paper a very strong resource for further studies. I appreciate that the present data is now also discussed with indications to certain phenomena (e.g. antiferroelectric ordering) rather that assigning the spectra as proof of such effects. The wide discussing really provides a broad consideration of these various aspects. I think that the paper will influence the thinking in the field and provide a solid basis for continuous investigations of these aspects. As such I think the manuscript should be published with the extensive revisions provided.
